# Impact of Natural Genetic Variation on Gene Expression Dynamics

**DOI:** 10.1371/journal.pgen.1003514

**Published:** 2013-06-06

**Authors:** Marit Ackermann, Weronika Sikora-Wohlfeld, Andreas Beyer

**Affiliations:** 1 Cellular Networks and Systems Biology, Biotechnology Center, TU Dresden, Dresden, Germany; 2 University of Cologne, Albertus-Magnus-Platz, Cologne, Germany; 3 Center for Regenerative Therapy Dresden, Dresden, Germany; University of Queensland, Australia

## Abstract

DNA sequence variation causes changes in gene expression, which in turn has profound effects on cellular states. These variations affect tissue development and may ultimately lead to pathological phenotypes. A genetic locus containing a sequence variation that affects gene expression is called an “expression quantitative trait locus” (eQTL). Whereas the impact of cellular context on expression levels in general is well established, a lot less is known about the cell-state specificity of eQTL. Previous studies differed with respect to how “dynamic eQTL” were defined. Here, we propose a unified framework distinguishing static, conditional and dynamic eQTL and suggest strategies for mapping these eQTL classes. Further, we introduce a new approach to simultaneously infer eQTL from different cell types. By using murine mRNA expression data from four stages of hematopoiesis and 14 related cellular traits, we demonstrate that static, conditional and dynamic eQTL, although derived from the same expression data, represent functionally distinct types of eQTL. While static eQTL affect generic cellular processes, non-static eQTL are more often involved in hematopoiesis and immune response. Our analysis revealed substantial effects of individual genetic variation on cell type-specific expression regulation. Among a total number of 3,941 eQTL we detected 2,729 static eQTL, 1,187 eQTL were conditionally active in one or several cell types, and 70 eQTL affected expression changes during cell type transitions. We also found evidence for feedback control mechanisms reverting the effect of an eQTL specifically in certain cell types. Loci correlated with hematological traits were enriched for conditional eQTL, thus, demonstrating the importance of conditional eQTL for understanding molecular mechanisms underlying physiological trait variation. The classification proposed here has the potential to streamline and unify future analysis of conditional and dynamic eQTL as well as many other kinds of QTL data.

## Author Summary

Complex physiological traits are affected through subtle changes of molecular traits like gene expression in the relevant tissues, which in turn are caused by genetic variation. A genetic locus containing a sequence variation affecting gene expression is called an expression quantitative trait locus (eQTL). Understanding the tissue and cell type specificity of eQTL effects is essential for revealing the molecular mechanisms underlying disease phenotypes. However, so far the cell-state dependence of eQTL is poorly understood. In order to systematically assess the importance of cell state-specific eQTL, we propose to distinguish static, conditional and dynamic eQTL and suggest strategies for mapping these eQTL classes. We applied our framework to mouse gene expression data from four hematopoietic stages and related cellular traits. The different eQTL classes, although derived from the same expression data, represent functionally distinct types of eQTL. Importantly, conditional eQTL are well correlated with relevant hematological traits. These findings emphasize the condition specificity of many regulatory relationships, even if the conditions under study are related. This calls for due caution when transferring conclusions about regulatory mechanisms across cell types or tissues. The proposed classification will also help to unravel dynamic behaviors in many other kinds of QTL data.

## Introduction

Natural genetic variation affects gene expression levels and thereby impacts on molecular and physiological phenotypes such as protein levels, cell morphology or disease phenotypes. In this respect, gene expression has proven instrumental as an intermediate phenotype from which conclusions about the emergence of high level traits can be drawn. A genetic locus containing a sequence variant that affects transcript levels of a gene is called an *expression quantitative trait locus* (eQTL). Studying eQTL has demonstrated its value for revealing the molecular mechanisms underlying disease associated SNPs, that were previously identified e.g. through genome wide association studies (GWAS) [1], [2]. Moreover, it has been shown that eQTL SNPs are more likely to be disease causing than random genetic loci [3] and can thus be used to prioritize genetic markers in GWAS.

Differences in mRNA expression levels caused by natural genetic variation can manifest themselves between individuals, populations, environments and, very importantly, between cell types and tissues (see [4], [5] and references therein). Since cells forming different tissues must have very different morphology, organization and function, distinct patterns of gene expression are required for each cell type. This variation of gene expression between cell types is under the influence of natural genetic variation. A number of studies (summarized in Table 1) compared eQTL across different cell types and tissues in mouse and human samples and report that 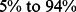 of the eQTL are cell type-specific. Potential reasons for the seemingly divergent outcomes of these studies are the different levels of relatedness of tissues under study and the different sample sizes of the studies. The last point is especially important in that cell type specificity is probably over-estimated due to low power of eQTL studies [4], [6]. Nevertheless, there is clear evidence for cross-tissue differences in genetic variation influencing transcript levels. This raises the question whether conclusions drawn from an eQTL study in one cell type or even a cell line translate to other cell types. The answer to this question is obviously relevant for explaining disease mechanisms with eQTL studies that are conducted in tissues other than the disease tissue or when several cell types are involved in the disease etiology [7]. Most diseases are caused by a limited set of highly specialized cells, but cell- and tissue interactions are crucial for their etiology. Understanding the tissue and cell type-specificity of molecular traits is therefore essential for revealing the molecular mechanisms underlying disease phenotypes.

**Table 1:** eQTL tissue specificity.

tissues	proportion of specific eQTL	ref	*cis*/*trans* eQTL	mapping strategy
liver, muscle, SAT, VAT, peripheral blood	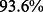	[74]	*cis*	separate mappings, meta-analysis on non-blood tissues
blood and LCL	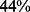	[75]	*cis*	separate heritability analyses
T helper and regulatory T cells	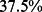 (*cis*), 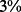 (*trans*)	[76]	*cis* and *trans*	separate mappings
blood and adipose tissue	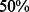	[77]	*cis*	single- and cross-tissue heritability estimates
LCL, skin and fat	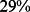	[5]	*cis*	separate mappings
normal, uninvolved and lesional psoriatic skin	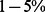	[78]	*cis*	separate mappings
liver, omental adipose and subcutaneous adipose tissue	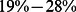	[3]	*cis* and *trans*	separate mappings; specificity defined as the fraction of eQTL occurring in at most 2 out of 3 tissues
LCL, heart, kidney, liver, lung, testes	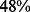	[79]	*cis*	separate mappings; eQTL were selected to have a strong effect
fibroblasts, LCL and T cells	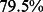	[4]	*cis*	separate mappings
HSC, myeloid progenitor cells, erythroid cells and myeloid cells	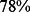	[10]	*cis* and *trans*	separate mappings, ANOVA including cell type and interaction effects
PBMC and cortical brain tissue	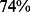	[80]	*cis*	separate mappings
blood and adipose tissue	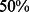	[81]	*cis*	separate mappings

Proportion of tissue-specific eQTL reported in different studies in mouse and human. We report the tissues/cell types that were analyzed, whether only local (i.e. *cis*) eQTL or both local and distant eQTL were inferred. The last column describes whether eQTL mapping was conducted separately in each cell type or by including a tissue factor into the analysis.

Another layer of complexity is added when considering dynamic processes such as cellular differentiation or responses to internal or external stimuli. These changes go along with drastic alterations of the cell's morphology or molecular state being induced through the adaptation of gene expression patterns. Therefore, it is important to not only compare eQTL observed in individual cell types (at steady state), but to additionally map the expression changes measured during cell state transitions. Intriguingly, the concepts of cell type-specific and differential eQTL have rarely been investigated together [8].

Hence, the main goals of the present study are to bring together and consolidate the different varieties of eQTL that have been proposed in the context of comparative eQTL mapping; to provide a thorough and functional classification of these eQTL classes reflecting the spectrum of genetic contributions to gene expression variation over a range of dynamically changing cell states; to show that these classes represent different sets of eQTL corresponding to different modes of expression variation and to demonstrate that their distinction facilitates the biological interpretation. A well-studied model for a dynamic process, being accompanied by substantial gene expression changes, is the differentiation of hematopoietic stem cells (HSC) into the different lineages of mature blood cells [9]. We decided to use this system to investigate eQTL based on three different categories of expression-based traits: (i) eQTL that are observed across all cellular states (*static eQTL*), (ii) eQTL being specific to one or a subset of cell states (*conditional eQTL*) and (iii) eQTL affecting changes of transcript levels during differentiation (*dynamic eQTL*). We propose strategies to map eQTL in the different classes and we demonstrate that eQTL from the above three classes, although based on the analysis of the same set of expression and genotype data, comprise different sets of regulatory loci having to be inferred from separate mappings. The choice of the eQTL mapping procedure has considerable influence on the outcome of the study. In particular, we show that basic cellular processes and state and differentiation specific functions are regulated by different eQTL categories. Although our scheme can serve to classify eQTL across any set of cell *states*, we will use the term cell *type* in the remainder of this paper, referring to the application to hematopoietic cell types.

## Results

### eQTL classification

**Figure 1:**
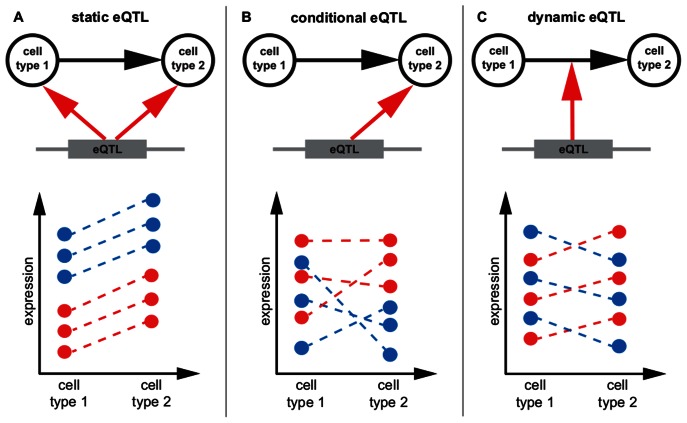
eQTL classification. Schematic representation of static, conditional and dynamic eQTL. For the sake of simplicity only two conditions are considered, but the concept is extensible to any number of cell types. The top part of each panel shows in which condition the eQTL influences a gene's expression (A, B) or if it affects expression changes between cell types (C). The lower parts of the panels show exemplary mRNA expression profiles of the gene in six samples. The genotype of the eQTL in each sample is indicated by the color, assuming homozygous diallelic markers. **A** A static eQTL impacts expression in all cell types. The ranking of gene expressions per genotype is the same in all conditions, as is the slope of expression change between cell types. **B** A conditional eQTL influences gene expression in only one of the two conditions. Thus, gene expression is a function of genotype in one cell type but not in the other. The slopes of expression changes may or may not be dependent on the genotype at the eQTL. **C** A dynamic eQTL drives expression changes between cell types. This implies that the slopes of expression changes between conditions are dependent on the genotype at the eQTL.

We distinguish static, conditional and dynamic eQTL (Figure 1, Table 2). A static eQTL affects a gene's expression in all conditions under consideration (Figure 1A). It is independent of the cell type and will thus be detected in all cell types. In contrast, a conditional eQTL can be found in one or a subset of the conditions under consideration (Figure 1B). In rare cases, a conditional eQTL might even be present in all four cell types. The difference between a static eQTL and a conditional eQTL active in all (i.e. four) cell types is the following: the static eQTL has the same effect throughout all cell types, whereas the conditional eQTL, although being active in all cell types, has effects dependent on the cell type. For example, the magnitude of the effect may differ between cell types or even the direction of the effect may change, i.e., the major allele may yield higher expression levels of the target gene in one cell type and lower expression levels in another cell type. A third reason for the cell type dependence of conditional eQTL is that the effect may be dependent on different co-factors, i.e. there might be different epistatic interactions with other markers dependent on the cell type.

**Table 2:** Overview of eQTL mapping methods.

Mapping method	Trait	Predictors
simultaneous mapping	concatenated gene expression over all cell types	genotypes, cell type indicators
single cell type mapping	gene expression in one specific cell type	genotypes
dynamic eQTL mapping	gene expression differences between a pair of cell types	genotypes

Overview of the traits and predictors of the eQTL mapping methods applied in this paper.

Both static and conditional eQTL impact the absolute expression levels of their target genes in the given cell types. As opposed to that, dynamic eQTL drive changes in mRNA levels during the transition from one cell type to another and thus act on expression differences between cell types (Figure 1C). Thus, the trait value used for mapping dynamic eQTL is the differential expression between two states or conditions (in other words, we use the fold-change between two conditions as a trait value).

In this respect our definition of dynamic eQTL differs from definitions used in the literature. For example, Gerrits et al. [10] define a dynamic eQTL as an eQTL that is present in one condition but not in another. We refer to those eQTL as conditional. A concept very similar to dynamic eQTL has been introduced in the context of studying transcriptional regulation in different growth conditions in yeast [8]. The authors define eQTL affecting expression changes between conditions as gene-environment interaction eQTL (gxeQTL). A similar study has been conducted on differential expression in two different temperatures in worms [11]. Despite their application by several groups, the three different eQTL classes have never been mapped and compared in one single study.

Different computational means can be used to detect the three eQTL types defined above. Dynamic eQTL require mapping of the expression changes (fold changes, slopes) observed at the transition from one type to another ([8], [11], Table 2, Methods Section “Dynamic eQTL mapping”). Conditional eQTL may be detected through independently mapping eQTL in the various cell types and then identifying such eQTL that were found in some, but not all conditions. Such an approach requires defining two thresholds: first a significance threshold (e.g. maximum p-value) for calling eQTL that are active in one cell type and second, an insignificance threshold (e.g. minimum p-value) for deciding that the same eQTL is not active in other cell types. Note that both thresholds are required and that they have to be sufficiently different. Using just one threshold would lead to a situation where all eQTL that are just above the threshold in one cell type and just below the threshold in other cell types would be called “conditional” although the eQTL scores are very similar across all conditions.

Here we propose a different approach that we termed “simultaneous mapping”, because it simultaneously identifies static and conditional eQTL and because it simultaneously uses the expression data from all conditions (Table 2).

### Simultaneous eQTL mapping

The goal of simultaneous eQTL mapping is to infer eQTL that are specific for each of the cell types under study (conditional eQTL) as well as static eQTL in one single analysis. Static eQTL should lead to expression patterns that are similar across conditions. Combining expression data from all conditions in a single mapping therefore drastically increases the statistical power for detecting static eQTL.

**Figure 2:**
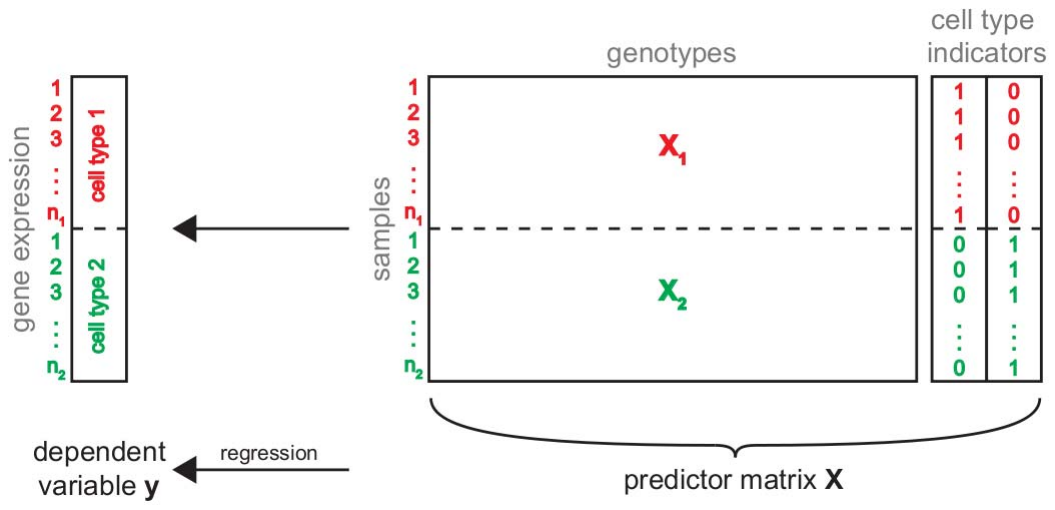
Simultaneous eQTL mapping. Schematic of simultaneous eQTL mapping for two cell types. This approach combines the available information from the two cell types (red and green) in one eQTL analysis. To this end, the gene expressions measured in the different conditions are combined into one vector 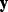. Similarly, for each condition the genotype matrix is subset to all samples for which there are expression measurements in this cell type. The resulting two submatrices 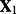 and 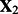 are concatenated into one genotype matrix. In order to discriminate static and conditional eQTL, two additional predictors indicating the cell type from which a sample was derived, are added to the predictor matrix. The combined genotype and cell type indicator matrix is used to find the model which best predicts gene expression simultaneously in all conditions.

To this end, we combined gene expressions over all cell types into one trait vector 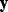 (Figure 2). This resulted in a single matrix containing the expression values of all genes and individuals across all conditions. In order to get a matching genotype matrix, we replicated the genotype matrix as many times as there are cell types. Because not all individuals (mouse lines) were measured under all conditions, we had to subset the genotype matrices to the samples for which gene expression data was available. The resulting matrices 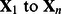 were concatenated in order to obtain a predictor matrix matching 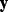. Finally, we added one new predictor for each cell type indicating whether a given sample belongs to the respective cell type. These additional variables allow to relate eQTL to the cell types in which they are active.

Next, the eQTL mapping was conducted using Random Forests (RF) [12], a multivariate machine learning technique that has been successfully tested on and applied to a number of QTL studies before [13]–[23] and that has been shown to outperform traditional univariate mapping approaches on simulated and real data [24]–[28]. RF learns decision trees based on bootstrap samples of the data. Genetic markers are used as predictor variables and RF will select markers if they are predictive for the expression of a given gene. Thus, the selection frequency can be used as a measure for the strength of an eQTL [25]. In case of static eQTL, a marker will be predictive of expression irrespective of the cell type. Thus, it will be predictive across the whole vector 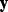. In the case of conditional eQTL, the marker will be predictive on only a subset of the samples, namely those corresponding to the cell type(s) in which the eQTL is present. Because the cell type indicator variables are part of the predictor matrix, RF can “split” the samples on such indicator variable and subsequently identify markers that are predictive for expression in the respective cell type. In both cases (static and conditional) such markers will have high selection frequencies, allowing them to be detected through appropriate permutation tests (Methods).

In order to determine if a significant eQTL is static or conditional we exploited interactions between markers and cell type indicators. Using ANOVA we tested if the predictive value of a marker depends on the cell type variable:
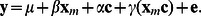
In this model, 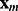 denotes the genotype vector of marker 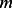, 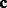 denotes the cell type factor variable with as many levels as there are types, 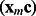 denotes their interaction and 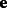 is a vector of normally distributed errors. The interaction term reflects the dependence of the eQTL on the respective cell type.

A static eQTL should not interact with the cell type variable since its activity is ubiquitous and does not depend on the cell type of the sample. On the other hand, conditional eQTL are active in one or a subset of the measured conditions and thus will show a significant interaction with the cell type in which they affect their targets. In this case, the model including the interaction term should explain the gene expression significantly better than a reduced model containing only main effects. If this is the case, i.e. if the False Discovery Rate (FDR) of the ANOVA is 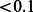, we call the eQTL “conditional”. Subsequent testing of contrasts can then identify the relevant cell types (Methods).

Overall, simultaneous eQTL mapping allows to discover static and conditional eQTL in one single analysis, thus reducing the multiple hypothesis testing problem as well as the computation time and rendering the choice for an “insignificance” threshold unnecessary. The approach of combining data over cell types also increases the power to detect static eQTL. Dynamic eQTL cannot be inferred with this approach since they are associated with a different trait, namely relative expression changes between cell types. Therefore, we analyzed dynamic eQTL in a separate mapping of gene expression differences using the same RF framework.

### Mouse hematopoiesis data

**Figure 3:**
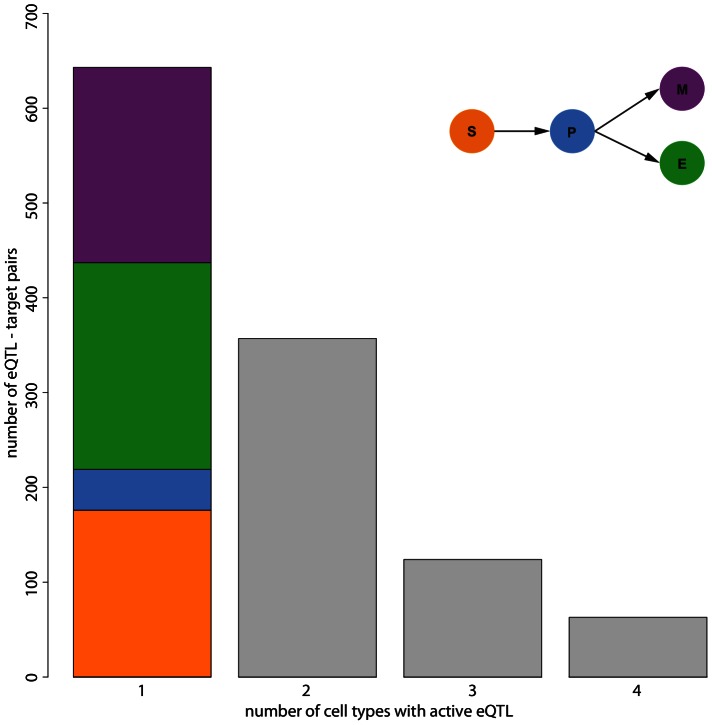
Number of cell types in which eQTL are active. The bars show the number of eQTL conditional in one, two, three or four cell types. Results are obtained from post-hoc Wald tests in the linear model comprising the eQTL marker, the cell type and their interaction. Only models with a significant marker - cell type interaction are considered. eQTL that are conditionally active in exactly one cell type are further classified by cell type (S - stem, P - progenitor, E - erythroid and M - myeloid cells).

Hematopoietic stem cell (HSC) differentiation is a prominent example of a dynamic process that is heavily genetically regulated [9], [29]–[32]. This has been shown, among others, by analyzing natural genetic variation between mouse recombinant inbred lines exhibiting very different hematopoietic phenotypes [33], [34]. One of the best studied examples is the panel of BXD recombinant inbred lines that were derived from crossing the C57BL/6 and DBA/2 lines. We are using genome-wide mRNA expression levels measured in 25 BXD strains in four cell types of HSC differentiation with varying degrees of lineage commitment: multipotent HSC with the potential for self-renewal, lineage restricted erythroid-myeloid progenitor cells, and lineage committed erythroid as well as myeloid cells (cf. scheme in upper right corner of Figure 3 and [10]).

We applied the above eQTL classification scheme to systematically search for genetic regions affecting gene expression dynamics during hematopoiesis as well as the static and conditional variation of expression in the different cell types. Using the data from [10], we focused on three cell type transitions during HSC differentiation: from stem to progenitor cells (S-P), from progenitor to erythroid cells (P-E) as well as from progenitor to myeloid cells (P-M). Prior to the analysis, we summarized the mRNA expression measurements to the gene level by calculating the median expression profiles across probes. After preprocessing (Methods) we selected 849 markers and expression data of 14,724 genes in 22 to 24 BXD strains per cell type.

### Frequencies of eQTL types

Our simultaneous eQTL mapping detected 3,916 significant eQTL target gene pairs at an FDR of 0.1. Among those, 2,729 eQTL did not show a significant interaction with the cell type indicator and thus constitute the class of static eQTL. We also found 1,187 conditional eQTL. These eQTL have to fulfill two conditions: (i) simultaneous mapping 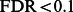 and (ii) FDR for interaction between marker and cell type indicator 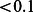. The majority of conditional eQTL was active (significant) in only one cell type (Figure 3). However, we also observed conditional eQTL being active in two, three, or even four cell types. eQTL with four significant cell type interactions arise if an eQTL is active in all cell types, but with changing effect sizes. Hence, a conditional eQTL active in four cell types is distinct from a static eQTL.

Most of the eQTL that are conditional in exactly one cell type (“cell type-specific”) occur in the more committed lineages (218 in erythroid cells, 206 in myeloid cells, Figure 3). We find less eQTL in the multipotent stem cells (176) and the smallest number of eQTL (43) in progenitor cells, an observation that is consistent with the original presentation of the data [10]. Likely, this reduced number of eQTL is due to increased levels of noise in the data, which in turn might be caused by different effects. First of all, purification of the cell types using FACS is imperfect. Thus, the observed expression levels actually reflect expression in a heterogeneous mix of cells. Increased impurity would then increase the level of noise and thus likely decrease the number of eQTL being detected. Another explanation comes from the fact that the progenitor cells are in a transient state. I.e., the dynamic nature of these cells might induce additional heterogeneity, which then also increases the noise and decreases the power to detect eQTL.

In contrast to the large number of static and conditional eQTL, we detected very few dynamic eQTL. At an FDR of 0.1 there were six eQTL driving gene expression changes during the transition from progenitor to erythroid cells and 66 eQTL for the transition from progenitor to myeloid cells. Two of the eQTL in these two groups are identical, i.e the same loci (both in *cis*) affect the same target genes during both, the P-E and the P-M transition. These targets are *Gadd45gip1* and *Lrrc51*. We were not able to find any dynamic eQTL in the transition from stem to progenitor cells.

**Figure 4:**
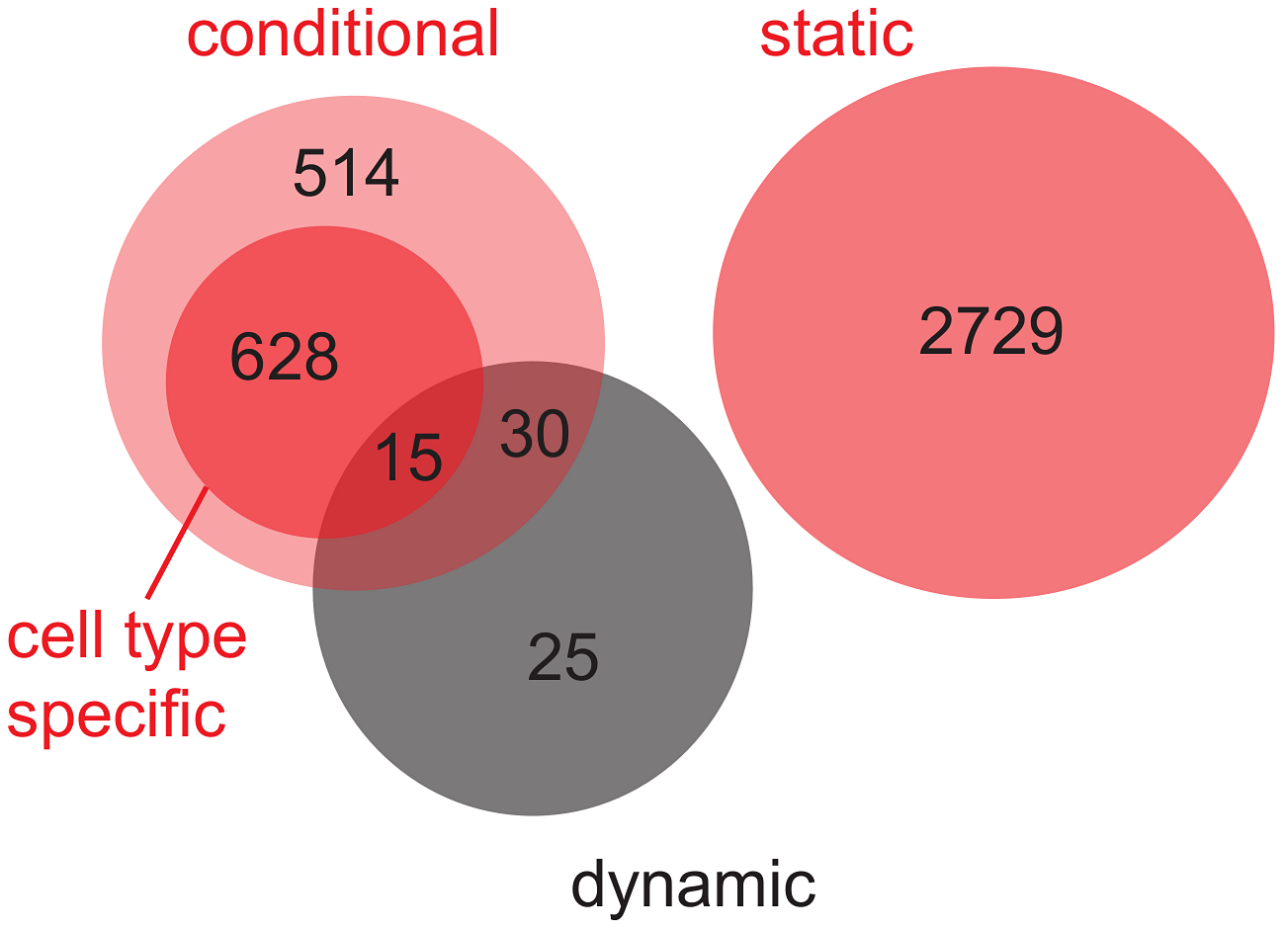
Venn diagram for the overlap between static, conditional and dynamic eQTL. Static and conditional eQTL were obtained from the simultaneous eQTL mapping (red circles). Cell type-specific eQTL (eQTL that are detected in exactly one cell type) are shown as a subgroup of conditional eQTL (dark red circle). Dynamic eQTL were derived from mapping expression differences between pairs of cell types (black circle). Results are summarized over the three cell type transitions that were analyzed (S-P, P-E, P-M).

Obviously, dynamic eQTL might overlap with conditional eQTL (“overlapping” means that the eQTL link the same locus-target gene pair, Figure 4). To facilitate comparison of conditional eQTL obtained with different mapping approaches (see Discussion), eQTL that are detected in exactly one cell type (i.e. cell type-specific eQTL) are shown as a subgroup of conditional eQTL. By definition, there is no overlap between conditional and static eQTL. As expected, none of the 70 dynamic eQTL overlap with static eQTL, while 45 coincide with conditional eQTL. Intriguingly, 25 loci that influence the dynamics of gene expression during the transition from one cell type to another (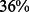 of all dynamic eQTL) could not have been detected by the simultaneous mapping, i.e. these eQTL did not overlap with eQTL from any other class [8].

### *cis*- versus *trans*-eQTL

An eQTL can either act locally (in *cis*) or on a distant gene (in *trans*). That is, the target gene of a *cis*-eQTL is encoded in the eQTL-region. A *trans*-eQTL refers to eQTL affecting a gene encoded elsewhere in the genome. Such influence can only be explained by *trans*-acting factors.

**Figure 5:**
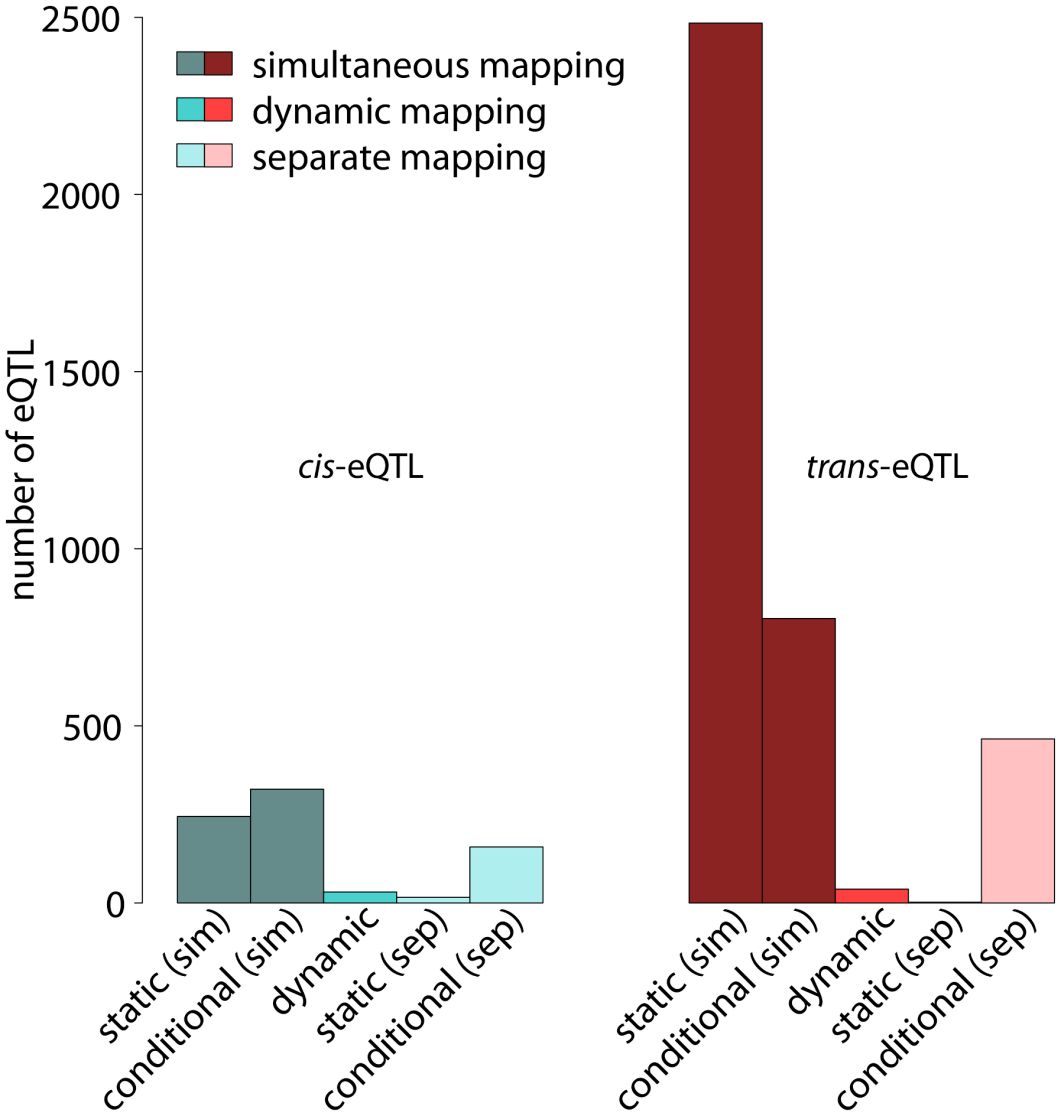
Number of *cis*- and *trans*-eQTL in different eQTL classes. Numbers of significant eQTL with 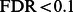 shown separately for cis-eQTL (left) and trans-eQTL (right). Static, conditional and dynamic eQTL are distinguished (see labels at the bottom). Further, the figure discriminates simultaneous and separate eQTL mappings, which represent alternative ways for distinguishing static and conditional eQTL. Simultaneous mapping increases the statistical power leading to substantially more eQTL significant at the same level (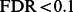). Even though both, *cis*- and *trans*-eQTL are increased when performing simultaneous mapping, *trans*-eQTL benefit more from the increase in power. See main text for exact definitions of the various eQTL types.

Around 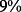 (244) of the static eQTL are *cis*-eQTL (left-hand side of Figure 5). It is noteworthy that the number of static and conditional *cis*-eQTL is relatively similar, whereas we find substantially more static than conditional *trans*-eQTL (Figure 5). The statistical power for detecting static eQTL is much higher than the power for detecting conditional eQTL in the framework of simultaneous eQTL mapping. This is because additional statistical power is needed for detecting significant differences between the cell types. References [10] and [35], among others, have shown that *cis*-eQTL are linked very strongly with their target genes while the effects of *trans*-eQTL are often weaker and several *trans*-eQTL are needed to explain the expression variation of a distant target gene. Hence, the increased power in case of static eQTL leads to an increased number of detectable *trans*-eQTL, whereas *cis*-eQTL seem to be “saturated” already at lower power. We confirmed this interpretation by varying the number of samples considered in the analysis, which showed that increasing the number of samples increased the number of detectable *trans*-eQTL more than the number of detectable *cis*-eQTL (Figure S1). This observation has two implications: first, the total number of *cis*-eQTL seems to be limited in this mouse population and second, it is possible to detect most *cis*-eQTL with a relatively small number of strains.

Dynamic eQTL comprise a much larger fraction of *cis*-eQTL compared to simultaneous eQTL (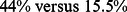, Figure 5). This is not surprising considering the fact that dynamic eQTL depend on gene expression measurements in two cell types at a time. They are thus more vulnerable to noise, but at the same time they have to be inferred from only one fourth of the samples available for the simultaneous mapping. Hence, we might only catch the strongest effects here, which are often found in *cis* [35].

### Comparison with the original analysis

**Figure 6:**
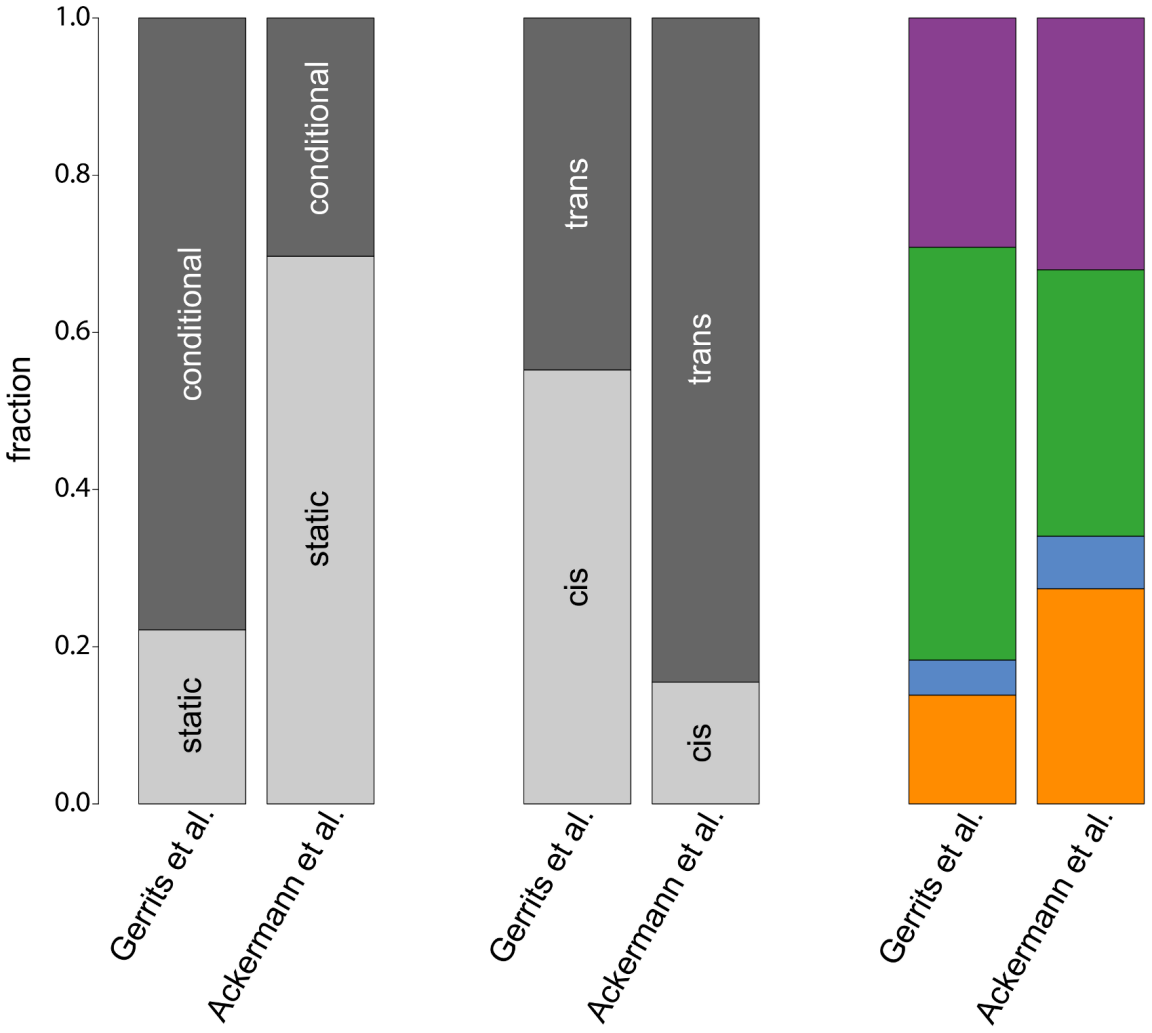
Comparison of eQTL analyses. The bars compare fractions of different eQTL classes obtained in the original study by [10] with our study. The leftmost bars show fractions of static and conditional eQTL, fractions of *cis*- and *trans*-eQTL are shown in the center. The rightmost bars compare fractions of cell type-specific eQTL in the four hematopoietic lineages (color scheme as in Figure 3).

A comparison of the results of our analysis with the original results from [10] reveals considerable differences between both studies (Figure 6), which are caused by the different mapping approaches. First of all, the simultaneous mapping in combination with RF is able to capture many more (probably small effect) eQTL than a linear model [27], [32], [33]. However, since [10] based their results on the number of probes having at least one significant eQTL and we are reporting significant eQTL-target gene pairs, and since the number of significant eQTL depends on the chosen p-value or FDR thresholds, we decided to compare fractions of eQTL classes instead of absolute numbers. We restricted the comparison to the static and conditional eQTL classes, since there is no equivalent to dynamic eQTL according to our definition in the original paper.

Our study detected a much larger fraction of static eQTL than the original paper (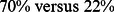) owing to the larger power of simultaneous mapping to capture this class of eQTL. Note that such ratios will always depend on the power to map eQTL in the corresponding classes with a given approach. Therefore, all ratios that have been reported so far (including our own) suffer from statistical biases. We cannot claim that any of them reflects “biological truth”.

Furthermore, the fraction of *trans*-eQTL is larger in our study compared to [10] (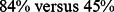, Figure 6, center). This can again be explained by the ability of the simultaneous mapping with RF to detect more small effect eQTL than a linear model.

In contrast, the fraction of cell type-specific eQTL from the four hematopoietic cell types is rather consistent between the two studies (Figure 6, rightmost bars). Interestingly, both studies detect only very few regulatory loci in progenitor cells, pointing to a general problem to detect specific regulatory relationships within this cell type. As mentioned before, this might be due to issues with the cell purification and the transient nature of this cell population.

### Cell type-specific eQTL-rich regions

**Figure 7:**
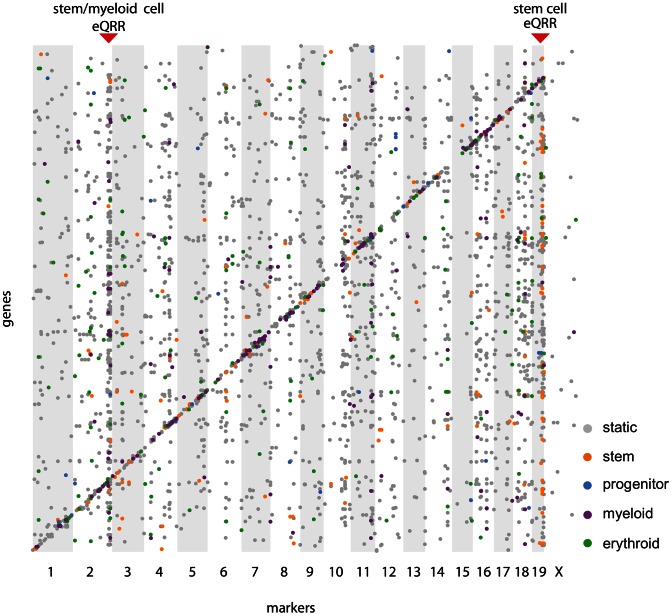
Simultaneous eQTL map. Each dot represents an eQTL - target gene pair, where physical marker positions are shown on the x-axis, gene positions on the y-axis. Significant static eQTL (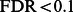) are shown in gray, cell type-specific eQTL (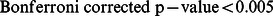 in exactly one cell type) are shown in the color scheme of Figure 3. Red triangles indicate two cell type-specific eQTL-rich regions (eQRR).

In order to show the conditionality of certain regulatory regions, we selected loci containing a larger number of eQTL-target pairs and tested their enrichment for conditional eQTL of a specific (subset of) cell types. This analysis is independent of the fact whether the given region has significantly more target genes than expected by chance as long as there are enough targets to be tested for conditionality. Therefore, we refer to these regions as “eQTL-rich regions”. The visualization of all cell type-specific and static eQTL in an eQTL map (Figure 7) reveals putative cell type-specific eQTL hotspots.

A Friedman test for differences in the distributions of contrast test p-values of the targets of such eQTL-rich regions uncovered some eQTL that have an effect on many genes in specific cell types. An example of such a hotspot is a locus on chromosome 19 (52.3–55.2 Mb) affecting 31 stem cell-specific and 59 static target genes. Even though only one third of the eQTL in this locus meet the significance threshold of a stem cell-specific eQTL, there is a clear tendency towards stem cell specificity for most of them (Figure S3A, 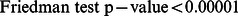). The eQTL contains the gene *Shoc2* for which we also find a *cis*-eQTL. We have previously shown that *trans* effects are often caused by genes being themselves affected through a *cis*-eQTL [36], which makes *Shoc2* a putative causal gene in the region. The protein encoded by this gene is a scaffold for a *Ras*/*Raf* interaction [37]. The *Ras* pathway is important for hematopoietic differentiation processes and frequently activated in hematopoietic malignancies [38]. However, we did not find any direct links between *Shoc2* and its putative target genes.

We found a second cell type-specific eQTL-rich region on chromosome 2 (168.3–169.7 Mb, 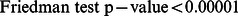), whose eQTL - target gene pairs are enriched for myeloid as well as stem cell-specific eQTL (Figure S3B). One possible regulator gene in this locus is *Nfatc2* (nuclear factor of activated T cells), which is gradually down-regulated at certain stages during the differentiation from myeloid progenitors to megakaryocytes and neutrophils [39]. Several of the eQTL target genes are predicted to be functionally related to *Nfatc2* [40] and many of them (e.g. *Ccdc99*, *Cdk2*, *Cdca8*, *Birc5*) are involved in cell cycle control. Indeed, it is known that *Nfatc2* negatively regulates the expression of *Cdk4*, which controls the entry and progression of a cell in the cell cycle [41]. In line with that, *Cdk4* links *Nfatc2* and its target genes in the STRING network. Although it has been shown that *Nfatc2* is not required to block cell cycle entry, it is likely that it prevents HSCs from differentiation into neutrophils and megakaryocytes via an effect on their proliferation [39], [42]. The importance of *Nfatc2* for both the HSC and the myeloid cells is reflected by the lower cell type specificity p-values of its targets in both types (Figure S3B) and corresponds well to *Nfatc2* expression levels that have been found to be high at the beginning of myeloid differentiation, go down during differentiation and finally increase again [39].

### Functional relevance of eQTL classes

**Figure 8:**
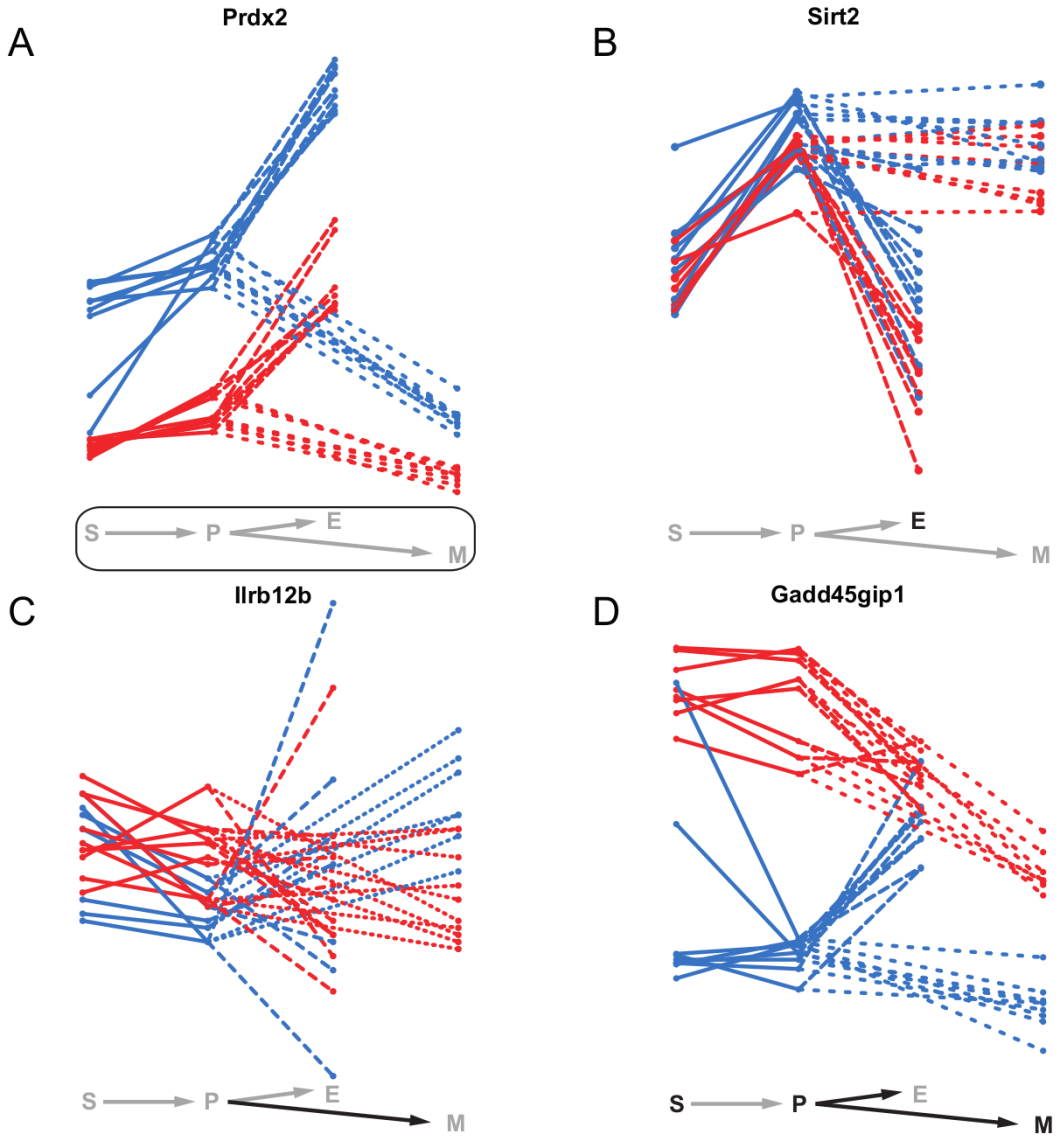
Examples of static, conditional and dynamic eQTL. mRNA expression profiles of four exemplary genes over the four hematopoietic cell types (S - stem cells, P - myeloid progenitor cells, E - erythroid cells, M - myeloid cells). The colors represent the genotype at the eQTL marker (blue - B allele, red - D allele). Significant static eQTL are shown by a rectangle around the differentiation scheme, significant conditional and dynamic eQTL by the black color of the respective cell type letter or cell type transition arrow. **A**, *Prdx2* is affected by a static eQTL in all four cell types. **B**, *Sirt2* is influenced by a conditional eQTL in erythroid cells. **C**, the transition of *Il12rb2* expression from progenitor to myeloid cells is driven by a dynamic eQTL. The expression of *Il12rb2* increases in samples carrying the B allele at the eQTL, while it remains constant in samples carrying the D allele. **D**, the expression of *Gadd45gip1* is conditionally affected in three of the four cell types (S, P and M) by an eQTL which at the same time also influences the gene's expression changes during the differentiation from progenitors to the erythroid and myeloid lineages.

Static eQTL affect a gene's expression in all cell types. Therefore, we expect their target genes to have different, broader biological functions than genes affected by non-static eQTL. An example of such a static eQTL is an eQTL impacting on the expression of Peroxiredoxin-2 (*Prdx2*) (Figure 8A), a gene involved in the response to and protection of erythrocytes against oxidative stress [43]. It is one of the most abundant proteins in erythrocytes [44], which is reflected in the elevated expression levels in erythroid cells compared to the other cell types. However, due to the severe impact of damage from oxidative stress on hematopoietic cell homeostasis in every cell type [45], *Prdx2* expression levels need to be controlled across all cell states. Since *Prdx2* is encoded at the same locus as the eQTL itself, the expression differences between the eQTL alleles are probably due to a mutation in the gene itself or in a *cis*-regulatory region.

Figure 8B shows the deacetylase *Sirt2* as an example of a gene being target of a conditional eQTL. The expression of *Sirt2* is strongly correlated with the alleles at the eQTL in erythroid cells, but not the other cell types. We found the expression of *Sirt2* to be correlated with hematocrit levels in female mice (data not shown). Thus, the eQTL indirectly affects hematocrit levels in mice through the regulation of *Sirt2*. In line with its elevated expression levels in myeloid cells, there is first evidence that *Sirt2* might be involved in myeloid differentiation [46].

*Il12rb2* is an example of a gene being affected by a dynamic eQTL. The gene encodes for a transmembrane protein constituting one subunit of the Interleukin 12 receptor complex. Together with other colony-stimulating factors Interleukin 12 is involved in myelo- as well as erythropoiesis [47], [48]. We find a dynamic eQTL for *Il12rb2* in the differentiation from progenitor to myeloid cells, which is characterized by almost constant expression levels for strains carrying the D allele at the eQTL while mRNA levels increase for individuals carrying the B allele. The expression profiles of *Il12rb2* in progenitor and myeloid cells indicate that the eQTL might actually be conditional in both cell types with very small and opposite effects. Hence, such switching allelic effects are an example of a situation where dynamic eQTL mapping has increased power compared to conditional mapping.

Intuitively, one expects that a significant allele-dependent expression change from one to another cell type (i.e. a dynamic eQTL) will coincide with significant, allele-dependent expression in at least one of the two cell types involved in the transition (i.e. a conditional eQTL). We often observed such coincidence (Figure 4) and the cell cycle inhibitor *Gadd45gip1* [49] is a particularly interesting example (Figure 8D). *Gadd45gip1* is one of only two genes for which we found a dynamic eQTL affecting the transition to both, erythroid and myeloid cells. The protein encoded by this gene physically interacts with *Gadd45b*, which is involved in cell growth arrest during myeloid cell differentiation [49], [50]. *Gadd45gip1* might support this function and arrest cell cycle in a particular phase in myeloid precursor cells, which is a prerequisite for differentiation [51]. *Gadd45gip1* is up-regulated in stem and progenitor cells in samples carrying the D allele at the eQTL locus (Figure 8D). The eQTL is in *cis*, suggesting that a mutation in the *Gadd45gip1* gene itself or in its promoter region leads to decreased expression of the gene in individuals carrying the B allele. Accordingly, down-regulation of *Gadd45gip1* during the transition to myeloid cells only occurs in samples carrying the D allele. This leads to a dynamic eQTL from progenitor to myeloid cells. Interestingly, individuals having high *Gadd45gip1* levels in progenitor cells show a down-regulation of its expression during the transition to erythroid cells, while the gene is up-regulated in individuals with low *Gadd45gip1* levels in progenitor cells. This leads to an expression equilibration in erythroid cells and to a dynamic eQTL. Thus, (i) compensatory feedback mechanisms can “revert” the effect of an eQTL and (ii) there seems to be a need to tightly control *Gadd45gip1* expression in erythroid cells.

In order to test more systematically whether cell type independent (i.e. static) eQTL impact on different cellular functions than conditional and dynamic eQTL, we assessed the enrichment of functional categories among genes causing eQTL and among genes being affected by eQTL using gene annotations obtained from Gene Ontology (GO) Biological Process [52]. Such GO enrichment analysis is non trivial for genetic regions causing eQTL, because these regions typically contain multiple genes and it is usually unknown which of them is the true causal gene [53]. Therefore, we decided to annotate each region with the GO terms of all associated genes (see Methods). This rigorous solution avoids false positive GO enrichment due to local clusters of functionally related genes. The enrichment testing was conducted with the R package topGO [54], which corrects for the nested structure of GO. Since we found only six significant dynamic eQTL for the differentiation towards erythroid cells, we did not perform GO enrichment for this subset of eQTL. The top 10 most significantly enriched GO terms for each eQTL mapping are reported in Tables S1, S2, S3, S4, S5, S6, S7, S8, S9, S10, S11, S12.

**Figure 9:**
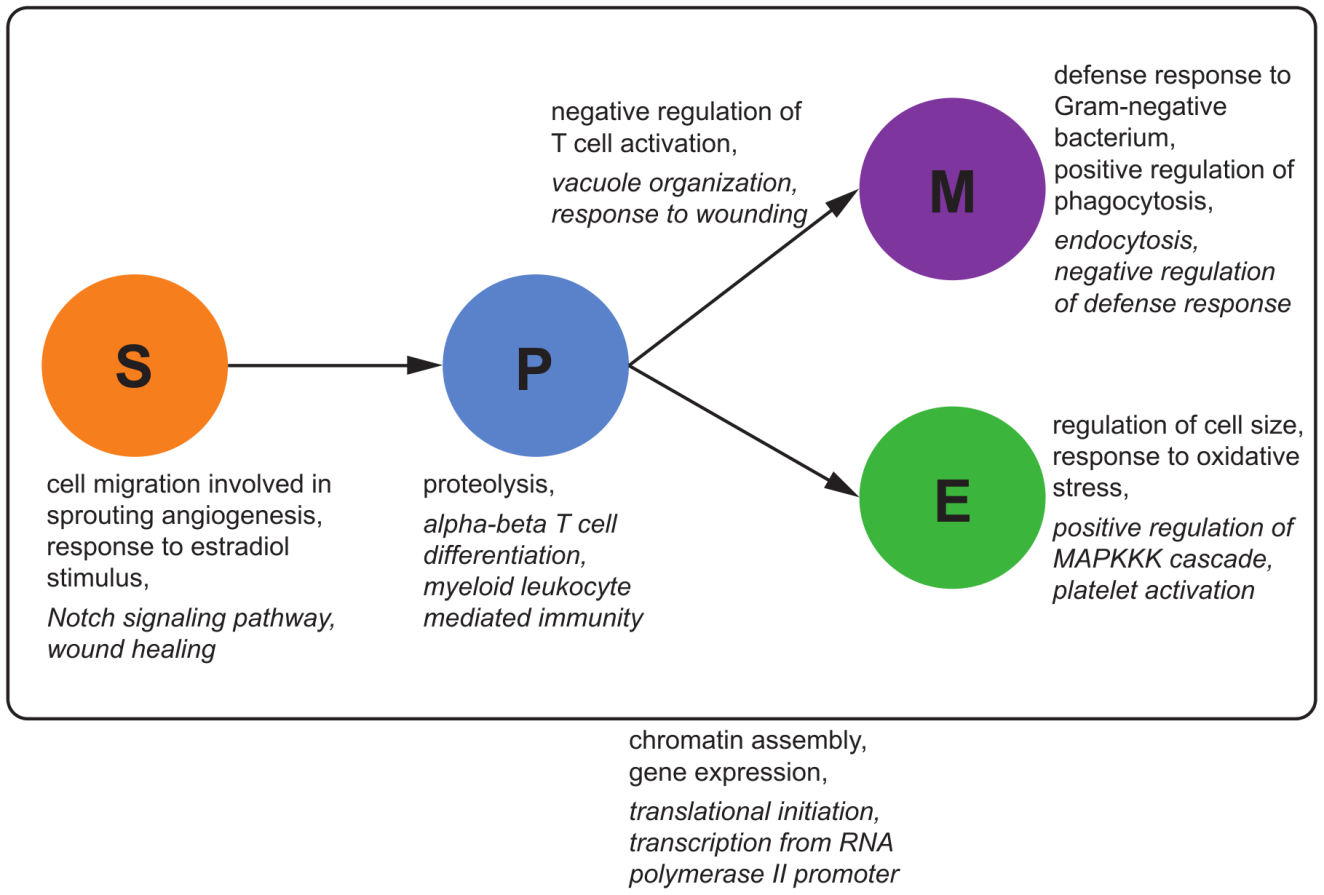
GO enrichment for eQTL classes. We tested for the enrichment of GO categories among eQTL loci and target genes in the different eQTL classes, separately for different cell types and transitions. Examples of enriched functional categories for cell type-specific and dynamic eQTL are shown next to the corresponding cell types or cell type transitions. Important GO categories that were enriched in static eQTL and their targets are shown outside the box. Terms that are significantly enriched (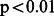) among eQTL loci are shown in italic, GO categories enriched among eQTL targets in regular font. See Tables S1, S2, S3, S4, S5, S6, S7, S8, S9, S10, S11, S12 for a list of the top significant GO terms of each mapping.

Figure 9 shows exemplary results of the enrichment distinguishing cell type-specific, dynamic and static eQTL. Static eQTL are enriched for very generic functional categories such as translation, transcription and cell cycle regulation. As opposed to that, conditional eQTL are enriched for hematopoiesis-related functions: For example, stem cell eQTL targets are enriched for the term “cell migration involved in sprouting angiogenesis”, in which HSCs play an important role [55]. Myeloid progenitor cell eQTL are enriched for the generic immune term “myeloid leukocyte mediated immunity”, while conditional eQTL in myeloid cells are enriched for very specific immune response terms like “defense response to Gram-negative bacterium”. We found several GO terms related to MAP kinases enriched among eQTL in erythroid and myeloid cells. This family of serine/threonine kinases plays a crucial role in maintenance and differentiation of HSC, especially during erythropoiesis [56].

Dynamic progenitor-myeloid eQTL are specifically enriched for categories related to T cell selection. This could be an indirect effect related to the role of macrophages and dendritic cells, which belong to the myeloid lineage, in adaptive immunity. These cells are involved in presenting antigens bound to the major histocompatibility complex (MHC) to naive T cells in order to activate or suppress these cells [57]. Accordingly, we find MHC coding genes among the dynamic eQTL targets. This analysis shows that static, conditional, and dynamic eQTL affect functionally distinct classes of genes and it therefore underlines the need to distinguish these types of eQTL.

### Improving the understanding of physiological traits

It has been suggested that eQTL might help better understanding the molecular mechanisms underlying the variation of physiological traits (i.e. causing “physiological QTL”). This notion is based on the observation that expression variation is underlying the variation of many physiological traits [7]. Indeed, eQTL studies have already demonstrated their value for the prioritization of disease associated SNPs [3], [58], [59]. Moreover, some of these studies have shown that there exists an association between the disease and the tissue in which the eQTL was found [5]. These findings suggest that knowledge about the eQTL class and (in the case of conditional and dynamic eQTL) the tissues in which it is detected might further improve our understanding of the molecular mechanisms causing the disease symptoms.

In order to investigate the impact of eQTL conditionality on physiological trait QTL, we analyzed the representation of different eQTL classes among QTL affecting hematological phenotypes (downloaded from www.genenetwork.org [60]). Out of 91 hematological phenotypes available, we selected 13 traits for which we found at least one significant QTL (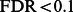) based on at least 15 BXD strains. In total, we found 17 QTL associated with those 13 physiological traits and further investigated all 15 of those with at least one significant eQTL linking to the corresponding QTL region (Tables S13, S14, S15, S16, S17, S18, S19, S20, S21, S22, S23, S24, S25, S26, S27). One QTL affected two very similar traits (“transferrin saturation of males and females” and “transferrin saturation of females”). Therefore, we counted it as one QTL in all subsequent analyses.

We found that the eQTL linking to these regions were enriched for *cis*-eQTL (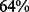 of the QTL and 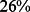 of all regions contain a *cis*-eQTL), which was associated with an increased number of conditional eQTL (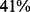 of all eQTL - target gene pairs in these loci were conditional, compared to 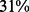 overall). The cell types in which these *cis*-eQTL were active, were often related to the respective cellular phenotype, suggesting that indeed these *cis*-eQTL are underlying the physiological changes. For example, we found five *cis*-eQTL in a region affecting hemoglobin levels in female mice (Table S13). Based on their known function, only two of the respective genes were plausible candidates for actually affecting hemoglobin levels: *E2f1* and *Asxl1* [61], [62], where the latter apparently has only very mild effects. Consistent with this, *E2f1* was the only gene among those five having a specific, conditional *cis*-eQTL in erythroid precursors, the cell type most closely related to the hemoglobin phenotype. Thus, the consideration of cell-type specific eQTL facilitates the identification of plausible candidate genes.

## Discussion

### Distinguishing static and non-static eQTL

The difference between static and non-static eQTL was very striking in our analysis. Due to the increased statistical power resulting from the simultaneous mapping we could identify substantially more static than non-static eQTL. Further, static and non-static eQTL differed substantially with respect to the functions of the involved genes, regarding both regulators (i.e. loci) and target genes. Whereas static eQTL involve mostly genes with generic, unspecific functions, non-static eQTL affect more cell type-specific pathways.

We found relatively few dynamic eQTL, ranging from zero (stem to progenitor cells) to 66 (progenitor to myeloid cells) per cell type transition. This is not very surprising given the fact that expression differences are prone to increased noise since they “inherit” the independent errors of expression experiments in two different conditions [63]. We would also expect a large overlap between conditional and dynamic eQTL. If there is a dependency between gene expression levels and genotype in one but not another cell type, then the magnitude of expression change between these cell types (i.e. the slope) should be genotype-dependent as well. However, we only find 45 eQTL as belonging to both, the conditional and the dynamic class, while 1,142 and 25 eQTL are exclusively conditional and dynamic, respectively.

One reason for this observation is the reduced power of the dynamic mapping leading to a failure to replicate conditional eQTL. Intriguingly, we also detect dynamic eQTL that we do not find among the conditional eQTL. Thus, there are modes of expression variation that are detectable with higher power when mapping expression differences instead of absolute expression levels. For example, we find eQTL with swapping effects on transcript levels (such as *Il12rb2*, Figure 8C) among 10 out of the 25 eQTL-target gene pairs that are unique in the dynamic class. This emphasizes the need to include different expression traits (like expression differences) into a comprehensive eQTL analysis in order to detect a wide spectrum of eQTL.

Another notable feature of dynamic eQTL mapping is its ability to mitigate systematic measurement errors affecting all cell types in a similar way. In this respect, a score for relative expression change can still be meaningful even though the absolute expression levels were not [63].

### Alternative eQTL mapping approaches

The approach we proposed for mapping different classes of eQTL is only one of a palette of possible strategies. Since the focus of the present work was on the introduction of a comprehensive, coherent and functional eQTL classification, in particular the discussion of each classes' characteristics and its implications on biological interpretation of eQTL results, we did not comprehensively compare different approaches for eQTL mapping. However, we still tested several variants, in particular the aggregation of static and conditional eQTL from separate mappings in every condition, which is the most widely used approach for comparative eQTL studies in the literature (see references in Table 1). Comparative eQTL studies have so far mostly mapped eQTL separately in each cell type, subsequently classifying eQTL as “static” if they are significant in all mappings, otherwise as “cell type-specific” (Table 1). This approach leads to a situation very different from our simultaneous mapping: in separate mappings an eQTL has to be significant independently in each cell type in order to be classified as static. In other words, large power is needed to detect static eQTL. As opposed to that, in our approach the eQTL has to be significantly dependent on the cell type in order to be classified as conditional. Therefore, simultaneous mapping is more conservative with respect to calling conditional eQTL. As a consequence, eQTL obtained with these two mapping strategies overlap only partially (Figure S2), which is mostly owed to the fact that simultaneous eQTL mapping detects many more significant eQTL, the largest fraction of which are static.

The advantage of the simultaneous mapping with Random Forests (combined with an ANOVA to disentangle conditional eQTL) instead of doing an ANOVA only is its ability to detect non-linear relationships. Therefore, the simultaneous mapping is able to detect a larger range of regulatory genetic variation than the simple linear model.

The strategy we followed for mapping dynamic eQTL has an obvious counterpart for static eQTL, namely the mapping of mean expression levels over all conditions. However, when applying this approach to the four hematopoietic cell types, we noticed that a large fraction of the resulting static eQTL were in fact conditional eQTL in one or several types. The erroneous classification resulted from the fact that a strong cell type-specific effect may dominate mean expression levels. Thus, this approach is prone to detect false positive static eQTL and in our opinion is not well suited to classify static eQTL.

### Consequences of cell type specificity for biomedical studies

The fact that we find 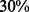 of all simultaneous eQTL to be conditional for one or several cell types emphasizes the condition specificity of many regulatory relationships, even if the conditions under study are very related. Note that simultaneous mapping is conservative for calling conditional eQTL and the true fraction of conditional eQTL is most likely even higher. In addition, we find that the number of conditional eQTL differs between cell types, partly due to differences in sample size and tissue impurity, but maybe also due to functional differences. The particular importance of conditional eQTL for cell type-specific molecular traits was further demonstrated by a GO enrichment analysis of eQTL and their targets in different eQTL classes. Moreover, an integration of eQTL results with QTL affecting hematological phenotypes revealed that a large fraction of these physiological QTL conditionally affect the expression of genes in phenotype-related cell types and are enriched for *cis*-eQTL.

It has previously been shown that eQTL causing variation of disease traits are often *cis*-eQTL [59]. Moreover, we and others have demonstrated that genes causing a *trans*-eQTL, i.e. affecting the expression of a distant target gene, often also exhibit a *cis*-eQTL affecting their own expression [9], [36]. Our analysis of the BXD mice confirms that genes with *cis*-eQTL are more likely causal. Beyond that, our results underscore the biomedical relevance of the distinction of different eQTL classes that we propose here, especially the impact of conditional eQTL on cell type-specific molecular and physiological phenotypes [58]. Since genetic variation affecting physiological phenotypes is often linked to conditional eQTL, the detection of the molecular mechanism underlying the QTL association critically relies on the cell type in which the eQTL study is conducted.

These findings call for due caution when drawing conclusions about regulatory mechanisms in one condition based on results from another condition [58], although other groups have claimed the innocuousness of such an approach [59]. A typical example for such a propagation of results would be the use of molecular mechanisms derived from eQTL studies in blood samples to explain disease mechanisms in other tissues like the brain [64]. The use of eQTL results for the elucidation of disease etiology is further complicated by the fact that the onset of complex diseases often involves pathways in several tissues.

Increasing statistical power by simultaneous mapping and distinguishing static, conditional and dynamic eQTL are important steps towards accounting for tissue and cell-type specificity, which is key for elucidating the molecular alterations underlying changes of complex physiological and disease traits [7].

### Generalization of the concepts

The classification of regulatory genetic variation is of course not limited to expression phenotypes. Almost all traits under genetic control (such as protein abundance, phosphorylation, alternative splicing and disease phenotypes, to name but a few) are dynamically regulated and depend on the specific context of the cell. Therefore, our classification scheme will be readily applicable to many other QTL studies and has the potential to unravel the dynamics underlying many biological processes. The simultaneous mapping will be beneficial to investigate different kinds of QTL across conditions and might even be extended (after appropriate data normalization) to comparative analyses across different datasets in the same organism.

## Materials and Methods

### Data processing

Preprocessed gene expression data of [10] were downloaded from GeneNetwork [60] (http://www.genenetwork.org, accession numbers GN144–151). The preprocessing comprised the 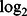 transformation and subsequent joint quantile normalization of expression data from all four cell types (HSCs, myeloid progenitors, erythroid and myeloid cells) as well as a batch correction. We mapped Illumina probe IDs to Ensembl gene IDs using mapping information from GeneNetwork and the R [65] biomaRt package [66] and summarized expression measurements for each gene by calculating the median expression profile over all its probes. Finally, we discarded all genes with a standard deviation of less than 0.1 in all four cell types, resulting in expression measurements of 14,724 genes on 22 to 24 BXD strains, depending on the cell type.

Genotype information of the BXD strains was also downloaded from GeneNetwork. Since we had expression information on only 25 strains, some neighboring genetic markers in the genotype matrix contained identical information (i.e. they were perfectly correlated). It is impossible to distinguish these markers with respect to their association to gene expression traits in the eQTL mapping. Therefore, we merged neighboring markers with identical genotype profiles across strains, which resulted in genotype information on 849 distinct markers or marker intervals across the mouse genome with a median interval size of 1.5 Mb (min: 4.6 kb, max: 32.1 Mb).

### Simultaneous eQTL mapping

To carry out eQTL mapping in all cell types simultaneously, each gene's expression vectors from all conditions are concatenated to form a new trait vector 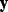 (Figure 2). Note that this vector might contain several entries for the same sample, each from a different cell type. Accordingly, genotype vectors belonging to each of the samples in each cell type are combined into a predictor matrix 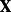. Since we would like to distinguish static and conditional eQTL, we need to add additional predictors indicating whether a sample was measured in a certain cell type or not. Therefore, 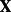 is extended by as many dummy variables as there are cell types.

We use Random Forests (RF) [12] for mapping eQTL. RF is a machine learning approach based on an ensemble of decision trees, each predicting gene expression for a different bootstrap sample of the data by testing different subsets of predictors at each split. We use the selection frequency of each predictor across the forest as a measure of its importance for predicting mRNA levels. A marker that is used more often than expected by chance is an eQTL of the corresponding gene. p-values are calculated using a permutation approach, see Subsection “p-value calculation”.

### Discrimination of static and conditional eQTL

For each significant eQTL - target gene pair (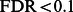), we fit two linear models to the gene expression: a full model containing the eQTL genotype, a cell type factor variable with as many levels as there are cell types and an interaction term between the two variables; and a reduced model containing only the two main effects without their interaction. If the full model explains the gene expression significantly better than the reduced one (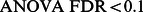), we call the eQTL “conditional”. The cell types in which the eQTL is active are found with post-hoc Wald tests ([67], chapter 1.3.3). The resulting p-values are corrected for multiple hypothesis testing using the stringent Bonferroni correction [68].

In principle, the second step of the simultaneous eQTL mapping, the distinction between conditional and static eQTL, could be directly resolved in the primary eQTL mapping step. The RF framework allows to extract epistatic interactions between predictors directly from the trees [16], [69]–[71]. However, this requires a large enough sample size in order to grow deep trees where different combinations of variables will be used for splitting in the same branch. When trying this line of action on the hematopoiesis data, it became clear that the small sample size (22 to 24 samples per cell type) is prohibitive for this step, leading to rather unstable results. Hence, we used the remedy of applying an ANOVA to filter the conditional eQTL out of the set of simultaneous eQTL. We believe that with the improvements made on costs and quality of large sequencing studies and the further increase in computing power this approach will become feasible very soon.

### Dynamic eQTL mapping

For mapping genetic loci driving expression dynamics between two cell types, we create a new trait vector containing the sample-wise expression differences of a given gene between the pair of cell types. The predictor matrix is made up of the marker genotype vectors of each sample for which expression changes could be inferred. We then conduct the eQTL mapping using RF as described for simultaneous eQTL in Subsection “Simultaneous eQTL mapping”.

### p-value calculation

We use the RF selection frequency (SF) as a measure of the impact of each genetic locus on gene expression. We have previously shown that this importance measure outperforms classic measures like the permutation importance in eQTL mapping [25]. However, the raw SF itself is not an absolute indicator of the importance of each predictor since the SF is biased for certain markers even under the null hypothesis [25].

A simple solution to this problem is the calculation of p-values based on a permutation approach: The expression vector is permuted many times. For each permutation, the eQTL mapping with the calculation of SFs is repeated. We assume that under the null hypothesis of no correlation between a given marker and a gene's expression, the distribution of SFs of that marker is the same for all genes. Hence, we pool SFs of each marker over all genes and all permutations in order to obtain a specific null distribution of SFs for each marker. Finally, the p-values of an eQTL - target gene pair are calculated as the fraction of permutation SFs exceeding the observed SF.

The bottleneck of this approach is the run-time of the RF, strongly restricting the number of permutations, which in turn results in a rather low resolution of the eQTL p-values, even after pooling SFs over genes. In order to overcome this problem, we decided to combine the permutation procedure with an analytical p-value calculation. After pooling SFs over a small number of permutations (10 in all our eQTL mappings), we fit an exponential function to the top one percent of the SF distribution. Consequently, we can calculate more precise p-values for the tail of the observed SF distribution, which contains the interesting eQTL - target gene pairs. The remaining 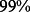 of the p-values are still obtained from the empirical SF distribution as described before. FDR is calculated with the procedure of Benjamini and Hochberg [72].

### GO enrichment analysis

We tested for the enrichment of certain biological functions among eQTL regions and target genes. We used Gene Ontology (GO) Biological Process [52] gene annotation, which we retrieved from the Ensembl database release 66 (www.ensembl.org) via the biomaRt [66] interface of R. eQTL loci were annotated with the functions of all genes encoded in the locus or being closer to this locus than to any other (if not more than 1 cM away from it). This approach ensures a conservative evaluation of functional enrichment and prevents a bias in the results due to clusters of functionally related genes within a locus. The GO enrichment testing was conducted using *topGO* [73] with the “weight” algorithm (R package topGO [54]). Although *topGO* already accounts to some extent for multiple hypothesis testing, we further calculated an empirical FDR for each term based on a shuffled gene/eQTL region to GO term assignment, preserving the number of terms assigned to each gene/region. We call all terms with an 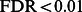 significant.

## Supporting Information

Figure S1 **Number of eQTL and proportion of** *cis* **-eQTL as a function of sample size.** We sub-sampled different numbers of strains in the simultaneous mapping (keeping ratios between cell types constant) and repeated the eQTL mapping. Panel A shows the number of eQTL in different classes as a function of sample size, while panel B shows the fraction of *cis*-eQTL among these. In order to detect any cell type-specific eQTL a minimum sample size larger than 20 is required. The proportion of *cis*-eQTL decreases with increasing sample size and is smallest for static eQTL, suggesting larger effect sizes for *cis*-eQTL compared to *trans*-eQTL.
(PDF)

Figure S2
**Comparison of different strategies for finding eQTL.** We compared the outcomes of three eQTL mapping approaches that are eligible to all or a subset of the eQTL classes. The Venn diagram shows the overlap between all the eQTL that were called significant in any of the mappings we used the method for. In particular, simultaneous eQTL are all eQTL with an 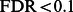 in the simultaneous mapping regardless of the ANOVA result. Dynamic eQTL had to be significant in at least one of the three cell type transitions (S-P, P-E, P-M) while cell type-specific eQTL were required to have an 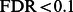 in at least one of the four cell types.(PDF)

Figure S3
**Distribution of contrast test p-values for cell type-specific eQTL hotspots.** eQTL hotspots might affect cell type-specific processes. This is shown for two eQTL-rich regions on chromosomes 19 (**A**) and 2 (**B**), respectively. Colors indicate hematopoietic cell types as in Figure 3. Overall, the stem (in **A**) and stem and myeloid cell (in **B**) contrast test p-values are much smaller than those for the other cell types, indicating that the marker locus is associated with the expression of genes involved in processes specific for the given cell type (p-values are shown in 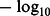 scale on the y-axis). The significance of the differences in contrast test p-values was assessed with Friedman's test, p-values are indicated in the top left corners.(PDF)

Table S1 **HSC specific eQTL targets.**
(PDF)

Table S2 **Progenitor specific eQTL targets.**
(PDF)

Table S3 **Erythroid specific eQTL targets.**
(PDF)

Table S4 **Myeloid specific eQTL targets.**
(PDF)

Table S5 **Dynamic progenitor to myeloid differentiation eQTL targets.**
(PDF)

Table S6 **Static eQTL targets.**
(PDF)

Table S7 **HSC specific eQTL markers.**
(PDF)

Table S8 **Progenitor specific eQTL markers.**
(PDF)

Table S9 **Erythroid specific eQTL markers.**
(PDF)

Table S10 **Myeloid specific eQTL markers.**
(PDF)

Table S11 **Dynamic progenitor to myeloid differentiation specific eQTL markers.**
(PDF)

Table S12 **Static eQTL markers.**
(PDF)

Table S13 **eQTL - target genes associated to the QTL of hemoglobin in females [g/dL].**
(PDF)

Table S14
**eQTL - target genes associated to the QTL of progenitor cell proliferation in young mice-effect of TGF-beta2 (0.1 ng/ml) on the proliferation of lin-Sca1++kit+ cells** 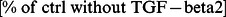 **.**
(PDF)

Table S15 **eQTL - target genes associated to the QTL of proliferative capacity in vitro of bone marrow stem and progenitor cells (lin-Sca1++ c-kit+ cells) in response to KL, flt3L and TPO [number of cells].**
(PDF)

Table S16
**eQTL - target genes associated to the QTL of transferrin saturation of males and females** 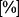 **.**
(PDF)

Table S17
**eQTL - target genes associated to the QTL of transferrin saturation of females** 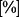 **.**
(PDF)

Table S18
**eQTL - target genes associated to the QTL of hemoglobin of 120-day-old females fed 270 ppm iron diet** 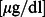 **.**
(PDF)

Table S19
**eQTL - target genes associated to the QTL of hemoglobin of 120-day-old females fed 270 ppm iron diet** 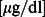 **.**
(PDF)

Table S20
**eQTL - target genes associated to the QTL of T cell receptor expression, V-gamma-1 positive, V-gamma-4 positive,** 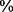 **of total gamma-delta intestinal intraepithelial lymphocytes** 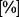 **.**
(PDF)

Table S21
**eQTL - target genes associated to the QTL of T cell receptor expression, V-gamma-7 positive and V-gamma-4] positive** 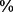 **of total gamma-delta intestinal intraepithelial lymphocytes** 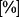 **.**
(PDF)

Table S22
**eQTL - target genes associated to the QTL of T cell receptor expression, V-gamma-7 positive and V-gamma-4] positive** 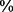 **of total gamma-delta intestinal intraepithelial lymphocytes** 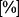 **.**
(PDF)

Table S23
**eQTL - target genes associated to the QTL of T cell receptor expression, V-gamma-7 positive, Vgamma-4 negative,** 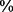 **of total gamma-delta intestinal intraepithelial lymphocytes** 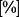 **.**
(PDF)

Table S24
**eQTL - target genes associated to the QTL of T cell receptor expression, V-gamma-7 positive, Vgamma-4 negative,** 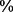 **of total gamma-delta intestinal intraepithelial lymphocytes** 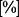 **.**
(PDF)

Table S25
**eQTL - target genes associated to the QTL of hematocrit of 120-day-old males and females fed 3 ppm iron diet** 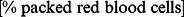 **.**
(PDF)

Table S26 **eQTL - target genes associated to the QTL of thymic T-cell response to anti-CD3-induced proliferation.**
(PDF)

Table S27
**eQTL - target genes associated to the QTL of iron level of plasma of 120-day male and female mice fed 3 ppm iron diet** 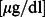 **.**
(PDF)
