# Supplementary figures and images for: Impact of Natural Genetic Variation on Gene Expression Dynamics
Source: PLoS Genet. 2013 Jun 6;9(6):e1003514. doi: 10.1371/journal.pgen.1003514 (PMC3674999; doi:10.1371/journal.pgen.1003514)

A

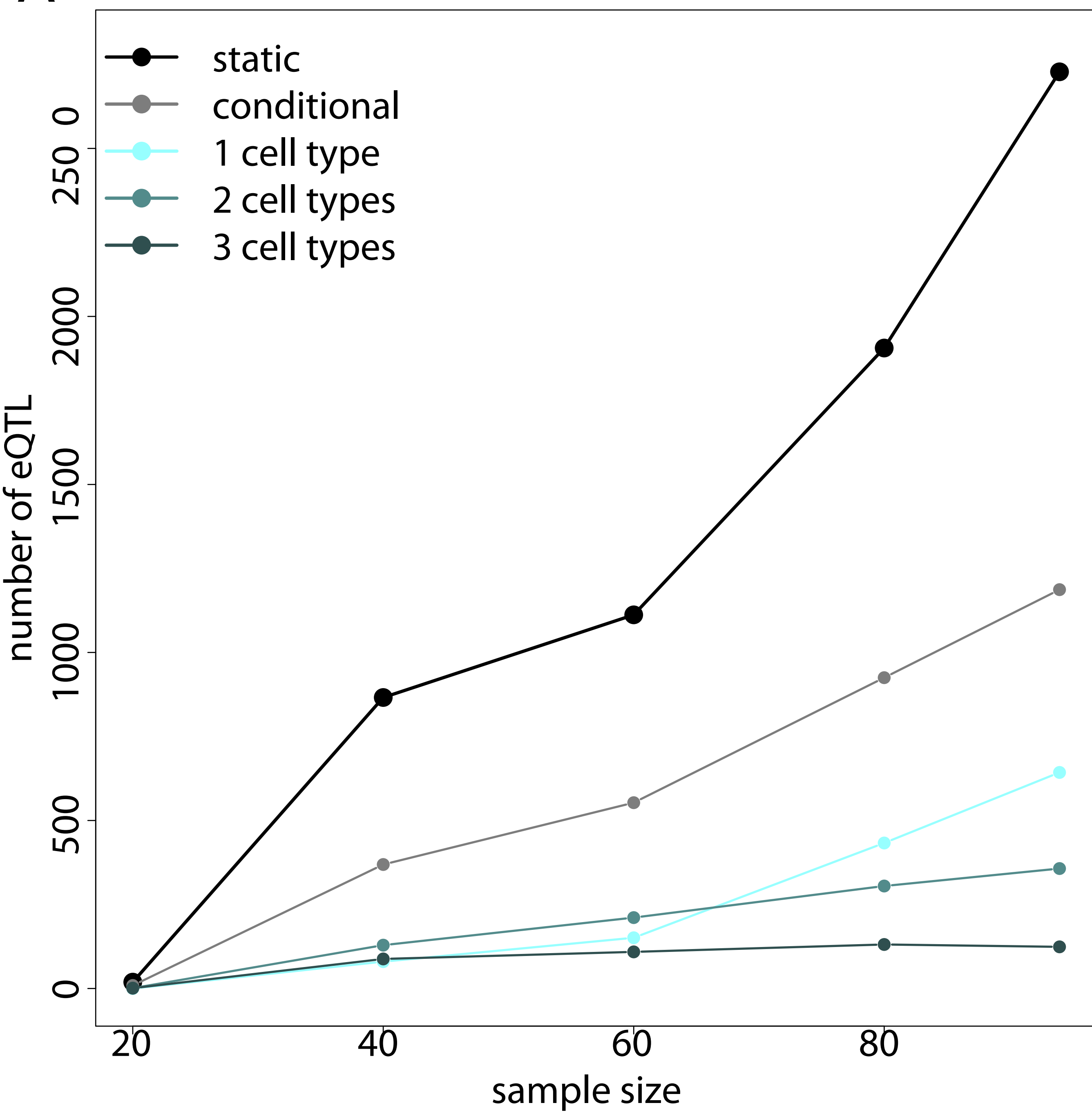

B

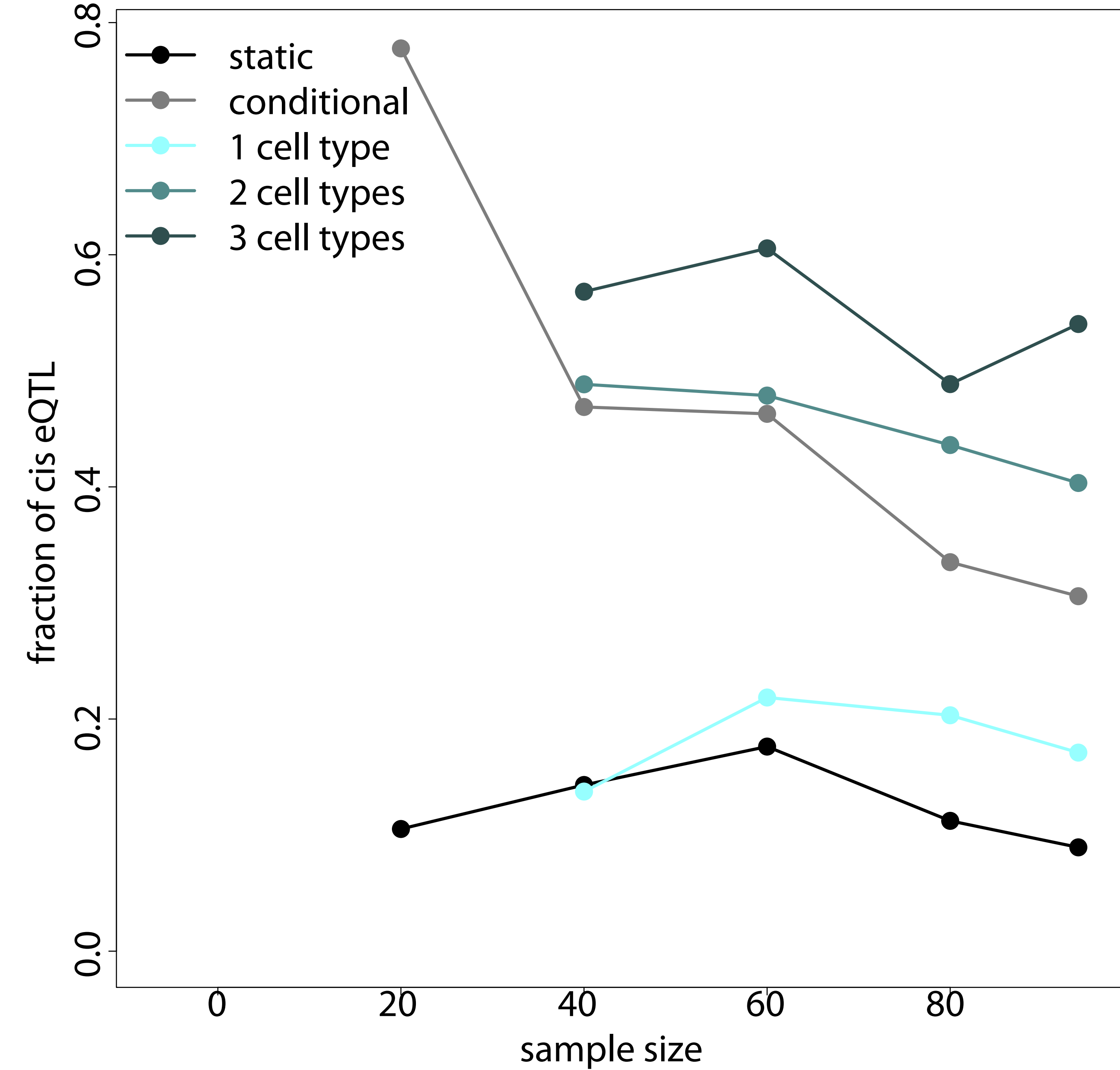

Supplement: Figure S1 — Number of eQTL and proportion of cis -eQTL as a function of sample size. We sub-sampled different numbers of strains in the simultaneous mapping (keeping ratios between cell types constant) and repeated the eQTL mapping. Panel A shows the number of eQTL in different classes as a function of sample size, while panel B shows the fraction of cis-eQTL among these. In order to detect any cell type-specific eQTL a minimum sample size larger than 20 is required. The proportion of cis-eQTL decreases with increasing sample size and is smallest for static eQTL, suggesting larger effect sizes for cis-eQTL compared to trans-eQTL. (PDF) [file pgen.1003514.s001.pdf]

simultaneous  
mapping

separate  
mapping

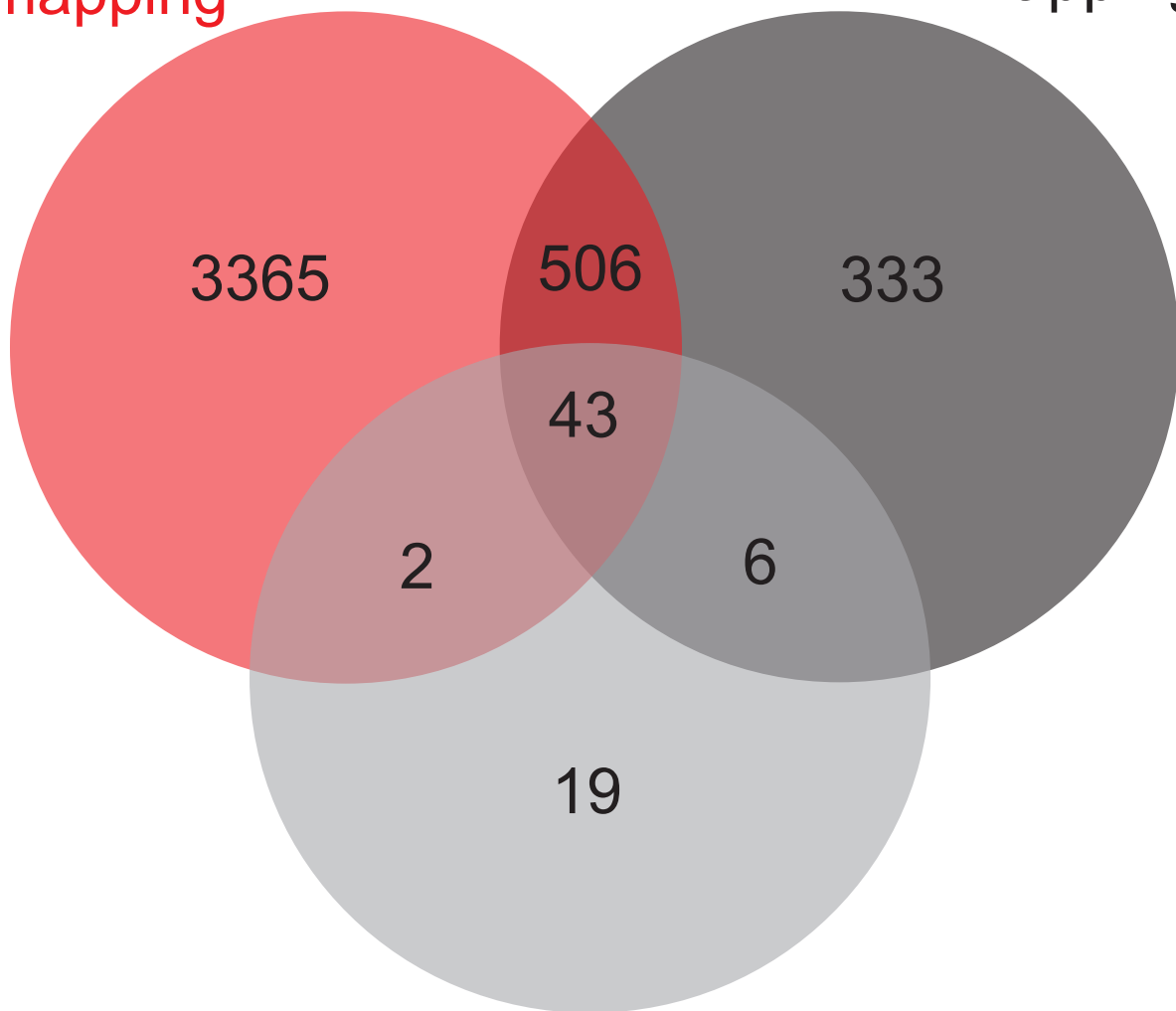

dynamic  
mapping

Supplement: Figure S2 — Comparison of different strategies for finding eQTL. We compared the outcomes of three eQTL mapping approaches that are eligible to all or a subset of the eQTL classes. The Venn diagram shows the overlap between all the eQTL that were called significant in any of the mappings we used the method for. In particular, simultaneous eQTL are all eQTL with an in the simultaneous mapping regardless of the ANOVA result. Dynamic eQTL had to be significant in at least one of the three cell type transitions (S-P, P-E, P-M) while cell type-specific eQTL were required to have an in at least one of the four cell types. (PDF) [file pgen.1003514.s002.pdf]

**A**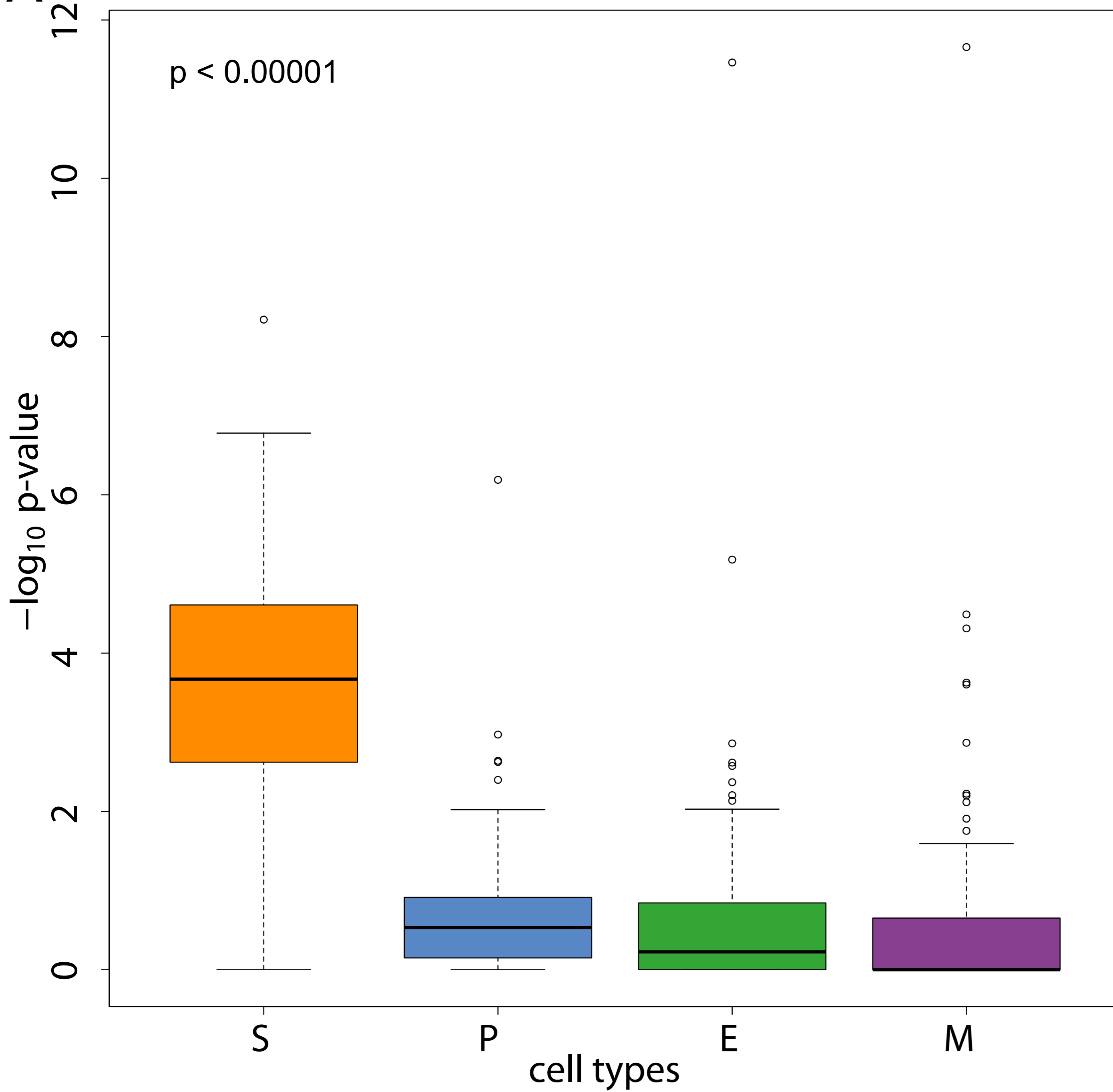**B**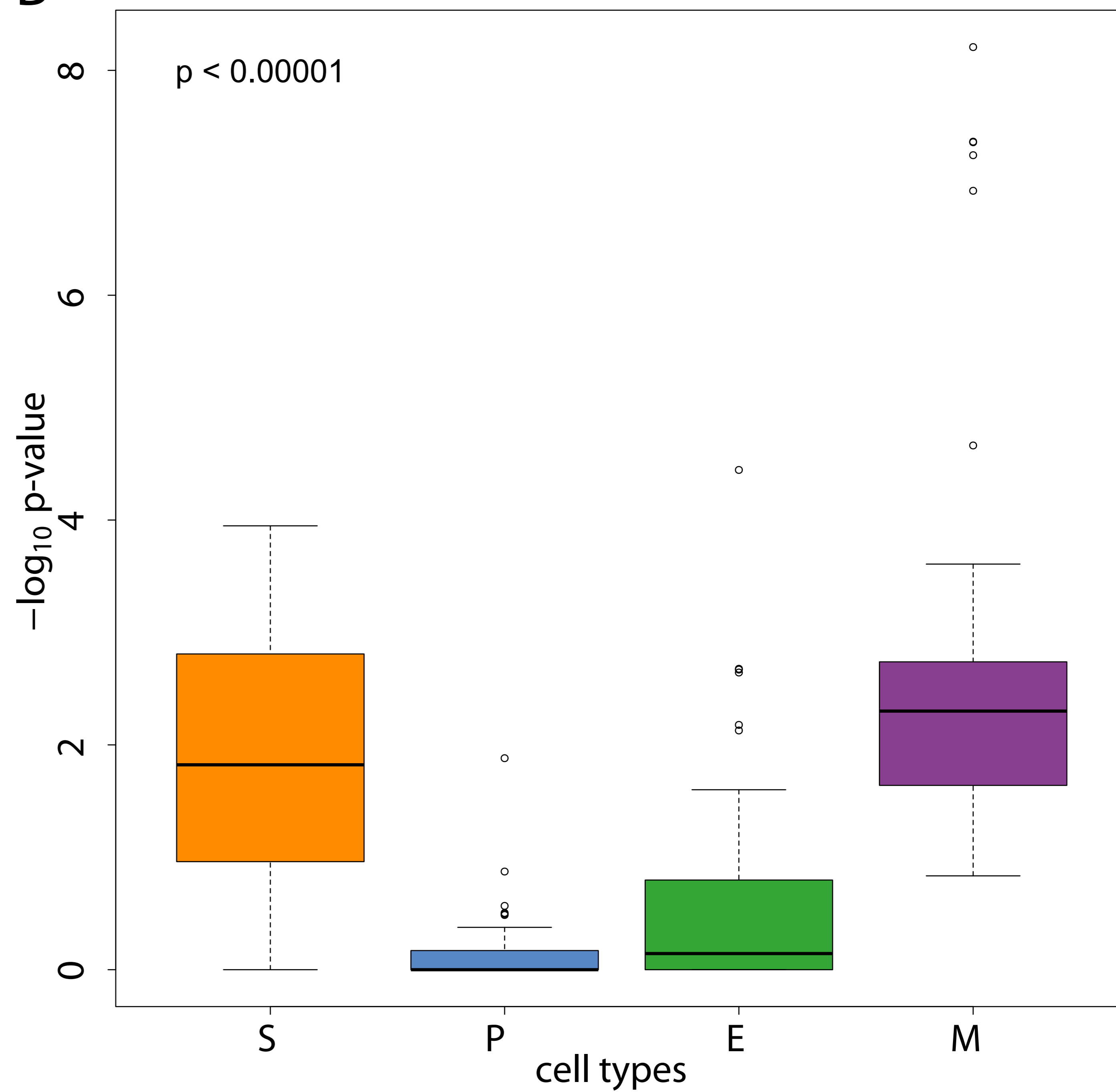

Supplement: Figure S3 — Distribution of contrast test p-values for cell type-specific eQTL hotspots. eQTL hotspots might affect cell type-specific processes. This is shown for two eQTL-rich regions on chromosomes 19 (A) and 2 (B), respectively. Colors indicate hematopoietic cell types as in Figure 3. Overall, the stem (in A) and stem and myeloid cell (in B) contrast test p-values are much smaller than those for the other cell types, indicating that the marker locus is associated with the expression of genes involved in processes specific for the given cell type (p-values are shown in scale on the y-axis). The significance of the differences in contrast test p-values was assessed with Friedman's test, p-values are indicated in the top left corners. (PDF) [file pgen.1003514.s003.pdf]
