# Supplementary material for: Impact of Natural Genetic Variation on Gene Expression Dynamics
Source: PLoS Genet. 2013 Jun 6;9(6):e1003514. doi: 10.1371/journal.pgen.1003514 (PMC3674999; doi:10.1371/journal.pgen.1003514)
Supplement: Table S1 — HSC specific eQTL targets. (PDF) [file pgen.1003514.s004.pdf]

Supplementary Table 1. HSC specific eQTL targets.

GO.ID	Term	p-value	FDR
GO:0001763	morphogenesis of a branching structure	0.00043	0.00028
GO:0002042	cell migration involved in sprouting angiogenesis	0.00043	0.00028
GO:0042036	negative regulation of cytokine biosynthetic process	0.00078	0.00055
GO:0032355	response to estradiol stimulus	0.00214	0.00111
GO:0000122	negative regulation of transcription from RNA polymerase II promoter	0.00214	0.00111
GO:0061039	ovum-producing ovary development	0.00495	0.00166
GO:0043406	positive regulation of MAP kinase activity	0.00541	0.00194
GO:0043112	receptor metabolic process	0.00616	0.00250
GO:0002688	regulation of leukocyte chemotaxis	0.00816	0.00333
