# Supplementary material for: Impact of Natural Genetic Variation on Gene Expression Dynamics
Source: PLoS Genet. 2013 Jun 6;9(6):e1003514. doi: 10.1371/journal.pgen.1003514 (PMC3674999; doi:10.1371/journal.pgen.1003514)
Supplement: Table S2 — Progenitor specific eQTL targets. (PDF) [file pgen.1003514.s005.pdf]

Supplementary Table 2. Progenitor specific eQTL targets.

GO.ID	Term	p-value	FDR
GO:0006508	proteolysis	0.0152	0.00056
