# Supplementary material for: Impact of Natural Genetic Variation on Gene Expression Dynamics
Source: PLoS Genet. 2013 Jun 6;9(6):e1003514. doi: 10.1371/journal.pgen.1003514 (PMC3674999; doi:10.1371/journal.pgen.1003514)
Supplement: Table S3 — Erythroid specific eQTL targets. (PDF) [file pgen.1003514.s006.pdf]

Supplementary Table 3. Erythroid specific eQTL targets.

GO.ID	Term	p-value	FDR
GO:0045744	negative regulation of G-protein coupled receptor protein signaling pathway	0.0017	0.00028
GO:0034341	response to interferon-gamma	0.0022	0.00056
GO:0006024	glycosaminoglycan biosynthetic process	0.0031	0.00056
GO:0045926	negative regulation of growth	0.0032	0.00083
GO:0006081	cellular aldehyde metabolic process	0.0041	0.00111
GO:0015807	L-amino acid transport	0.0041	0.00111
GO:0030166	proteoglycan biosynthetic process	0.0046	0.00111
GO:0008361	regulation of cell size	0.0070	0.00139
GO:0006979	response to oxidative stress	0.0088	0.00278
GO:0043066	negative regulation of apoptosis	0.0101	0.00305
