# Supplementary material for: Impact of Natural Genetic Variation on Gene Expression Dynamics
Source: PLoS Genet. 2013 Jun 6;9(6):e1003514. doi: 10.1371/journal.pgen.1003514 (PMC3674999; doi:10.1371/journal.pgen.1003514)
Supplement: Table S4 — Myeloid specific eQTL targets. (PDF) [file pgen.1003514.s007.pdf]

Supplementary Table 4. Myeloid specific eQTL targets.

GO.ID	Term	p-value	FDR
GO:0070838	divalent metal ion transport	0.00034	0.00083
GO:0050829	defense response to Gram-negative bacterium	0.00083	0.00139
GO:0050830	defense response to Gram-positive bacterium	0.00136	0.00194
GO:0030318	melanocyte differentiation	0.00171	0.00222
GO:0009132	nucleoside diphosphate metabolic process	0.00197	0.00222
GO:0050730	regulation of peptidyl-tyrosine phosphorylation	0.00266	0.00222
GO:0006611	protein export from nucleus	0.00384	0.00250
GO:0006886	intracellular protein transport	0.00419	0.00250
GO:0050766	positive regulation of phagocytosis	0.00480	0.00250
GO:0009166	nucleotide catabolic process	0.00497	0.00250
