# Supplementary material for: Impact of Natural Genetic Variation on Gene Expression Dynamics
Source: PLoS Genet. 2013 Jun 6;9(6):e1003514. doi: 10.1371/journal.pgen.1003514 (PMC3674999; doi:10.1371/journal.pgen.1003514)
Supplement: Table S5 — Dynamic progenitor to myeloid differentiation eQTL targets. (PDF) [file pgen.1003514.s008.pdf]

Supplementary Table 5. Dynamic progenitor to myeloid differentation eQTL targets.

GO.ID	Term	p-value	FDR
GO:0003229	ventricular cardiac muscle tissue development	0.00036	0.00000
GO:0003208	cardiac ventricle morphogenesis	0.00108	0.00000
GO:0050868	negative regulation of T cell activation	0.00164	0.00000
GO:0018108	peptidyl-tyrosine phosphorylation	0.02375	0.00083
GO:0006954	inflammatory response	0.02852	0.00083
