# Supplementary material for: Impact of Natural Genetic Variation on Gene Expression Dynamics
Source: PLoS Genet. 2013 Jun 6;9(6):e1003514. doi: 10.1371/journal.pgen.1003514 (PMC3674999; doi:10.1371/journal.pgen.1003514)
Supplement: Table S6 — Static eQTL targets. (PDF) [file pgen.1003514.s009.pdf]

Supplementary Table 6. Static eQTL targets.

GO.ID	Term	p-value	FDR
GO:0034645	cellular macromolecule biosynthetic process	< 0.00001	0.00000
GO:0034728	nucleosome organization	< 0.00001	0.00000
GO:0032774	RNA biosynthetic process	< 0.00001	0.00000
GO:0031497	chromatin assembly	< 0.00001	0.00000
GO:0010467	gene expression	0.00016	0.00000
GO:0006338	chromatin remodeling	0.00038	0.00000
GO:0016071	mRNA metabolic process	0.00049	0.00000
GO:0007034	vacuolar transport	0.00101	0.00027
GO:0000184	nuclear-transcribed mRNA catabolic process	0.00133	0.00082
GO:0002224	toll-like receptor signaling pathway	0.00133	0.00082
