# Supplementary material for: Impact of Natural Genetic Variation on Gene Expression Dynamics
Source: PLoS Genet. 2013 Jun 6;9(6):e1003514. doi: 10.1371/journal.pgen.1003514 (PMC3674999; doi:10.1371/journal.pgen.1003514)
Supplement: Table S7 — HSC specific eQTL markers. (PDF) [file pgen.1003514.s010.pdf]

Supplementary Table 7. HSC specific eQTL markers.

GO.ID	Term	p-value	FDR
GO:0007219	Notch signaling pathway	< 0.00001	0.00000
GO:0046394	carboxylic acid biosynthetic process	0.00003	0.00026
GO:0030641	regulation of cellular pH	0.00026	0.00184
GO:0006024	glycosaminoglycan biosynthetic process	0.00031	0.00184
GO:0042060	wound healing	0.00034	0.00184
GO:0070232	regulation of T cell apoptosis	0.00059	0.00236
GO:0060603	mammary gland duct morphogenesis	0.00066	0.00263
GO:0035108	limb morphogenesis	0.00073	0.00289
GO:0050817	coagulation	0.00076	0.00289
GO:0000266	mitochondrial fission	0.00076	0.00289
