# Supplementary material for: Impact of Natural Genetic Variation on Gene Expression Dynamics
Source: PLoS Genet. 2013 Jun 6;9(6):e1003514. doi: 10.1371/journal.pgen.1003514 (PMC3674999; doi:10.1371/journal.pgen.1003514)
Supplement: Table S8 — Progenitor specific eQTL markers. (PDF) [file pgen.1003514.s011.pdf]

Supplementary Table 8. Progenitor specific eQTL markers.

GO.ID	Term	p-value	FDR
GO:0046632	alpha-beta T cell differentiation	0.00029	0.00079
GO:0002444	myeloid leukocyte mediated immunity	0.00030	0.00079
GO:0032102	negative regulation of response to external stimulus	0.00077	0.00131
GO:0045604	regulation of epidermal cell differentiation	0.00078	0.00158
GO:0002456	T cell mediated immunity	0.00085	0.00158
GO:0048041	focal adhesion assembly	0.00091	0.00158
GO:0030334	regulation of cell migration	0.00096	0.00158
GO:0045807	positive regulation of endocytosis	0.00110	0.00158
GO:0051797	regulation of hair follicle development	0.00167	0.00210
GO:0006909	phagocytosis	0.00203	0.00263
