# Supplementary material for: Impact of Natural Genetic Variation on Gene Expression Dynamics
Source: PLoS Genet. 2013 Jun 6;9(6):e1003514. doi: 10.1371/journal.pgen.1003514 (PMC3674999; doi:10.1371/journal.pgen.1003514)
Supplement: Table S9 — Erythroid specific eQTL markers. (PDF) [file pgen.1003514.s012.pdf]

Supplementary Table 9. Erythroid specific eQTL markers.

GO.ID	Term	p-value	FDR
GO:0051091	positive regulation of sequence-specific DNA-binding transcription factor activity	0.00001	0.00053
GO:0043410	positive regulation of MAPKKK cascade	0.00002	0.00053
GO:0010883	regulation of lipid storage	0.00002	0.00053
GO:0071901	negative regulation of protein serine/threonine kinase activity	0.00007	0.00158
GO:0090207	regulation of triglyceride metabolic process	0.00007	0.00158
GO:0030168	platelet activation	0.00008	0.00184
GO:0031331	positive regulation of cellular catabolic process	0.00009	0.00236
GO:0043409	negative regulation of MAPKKK cascade	0.00010	0.00236
GO:0048610	cellular process involved in reproduction	0.00012	0.00263
GO:0051346	negative regulation of hydrolase activity	0.00012	0.00263
