# Supplementary material for: Impact of Natural Genetic Variation on Gene Expression Dynamics
Source: PLoS Genet. 2013 Jun 6;9(6):e1003514. doi: 10.1371/journal.pgen.1003514 (PMC3674999; doi:10.1371/journal.pgen.1003514)
Supplement: Table S10 — Myeloid specific eQTL markers. (PDF) [file pgen.1003514.s013.pdf]

Supplementary Table 10. Myeloid specific eQTL markers.

GO.ID	Term	p-value	FDR
GO:0006897	endocytosis	< 0.00001	0.00000
GO:0090207	regulation of triglyceride metabolic process	< 0.00001	0.00053
GO:0051222	positive regulation of protein transport	< 0.00001	0.00184
GO:0051052	regulation of DNA metabolic process	< 0.00001	0.00184
GO:0043407	negative regulation of MAP kinase activity	< 0.00001	0.00263
GO:0046889	positive regulation of lipid biosynthetic process	< 0.00001	0.00263
GO:0032102	negative regulation of response to external stimulus	< 0.00001	0.00263
GO:0031348	negative regulation of defense response	0.00002	0.00315
GO:0043409	negative regulation of MAPKKK cascade	0.00002	0.00420
GO:0043623	cellular protein complex assembly	0.00002	0.00420
