# Supplementary material for: Impact of Natural Genetic Variation on Gene Expression Dynamics
Source: PLoS Genet. 2013 Jun 6;9(6):e1003514. doi: 10.1371/journal.pgen.1003514 (PMC3674999; doi:10.1371/journal.pgen.1003514)
Supplement: Table S11 — Dynamic progenitor to myeloid differentiation specific eQTL markers. (PDF) [file pgen.1003514.s014.pdf]

Supplementary Table 11. Dynamic progenitor to myeloid differentiation specific eQTL markers.

GO.ID	Term	p-value	FDR
GO:0007033	vacuole organization	< 0.00001	0.00000
GO:0006644	phospholipid metabolic process	< 0.00001	0.00026
GO:0046677	response to antibiotic	0.00001	0.00079
GO:0045061	thymic T cell selection	0.00002	0.00079
GO:0016049	cell growth	0.00003	0.00105
GO:0017156	calcium ion-dependent exocytosis	0.00006	0.00184
GO:0009611	response to wounding	0.00006	0.00184
GO:0048813	dendrite morphogenesis	0.00006	0.00184
GO:0071229	cellular response to acid	0.00006	0.00184
GO:0071418	cellular response to amine stimulus	0.00006	0.00184
