# Supplementary material for: Impact of Natural Genetic Variation on Gene Expression Dynamics
Source: PLoS Genet. 2013 Jun 6;9(6):e1003514. doi: 10.1371/journal.pgen.1003514 (PMC3674999; doi:10.1371/journal.pgen.1003514)
Supplement: Table S12 — Static eQTL markers. (PDF) [file pgen.1003514.s015.pdf]

Supplementary Table 12. Static eQTL markers.

GO.ID	Term	p-value	FDR
GO:0032269	negative regulation of cellular protein metabolic process	< 0.00001	0.00026
GO:0006413	translational initiation	< 0.00001	0.00026
GO:0016311	dephosphorylation	< 0.00001	0.00026
GO:0009101	glycoprotein biosynthetic process	0.00001	0.00026
GO:0006417	regulation of translation	0.00002	0.00026
GO:0014070	response to organic cyclic compound	0.00002	0.00026
GO:0006366	transcription from RNA polymerase II promoter	0.00005	0.00131
GO:0009890	negative regulation of biosynthetic process	0.00007	0.00158
GO:0060070	canonical Wnt receptor signaling pathway	0.00010	0.00236
GO:0001892	embryonic placenta development	0.00011	0.00289
