# Supplementary material for: Impact of Natural Genetic Variation on Gene Expression Dynamics
Source: PLoS Genet. 2013 Jun 6;9(6):e1003514. doi: 10.1371/journal.pgen.1003514 (PMC3674999; doi:10.1371/journal.pgen.1003514)
Supplement: Table S13 — eQTL - target genes associated to the QTL of hemoglobin in females [g/dL]. (PDF) [file pgen.1003514.s016.pdf]

Supplementary Table 13. eQTL - target genes associated to the QTL of hemoglobin in females [g/dL].

Target gene	simultaneous FDR	ANOVA FDR	# sign. cond. eQTL	HSC p-value	progenitor cell p-value	erythroid cell p-value	myeloid cell p-value	P-M dynamic eQTL FDR	cis
Kif3b	< 0.00001	0.00005	4	< 0.00001	< 0.00001	0.00046	< 0.00001		yes
Cdk5rap1	0.00072	0.08611	3	< 0.00001	0.01980	< 0.00001	< 0.00001		yes
E2f1	0.04031	0.07424	1	0.00754	0.01779	0.00105	1		yes
Chmp4b	< 0.00001	0.58538	0						yes
Asxl1	< 0.00001	0.28660	0						yes
