# Supplementary material for: Impact of Natural Genetic Variation on Gene Expression Dynamics
Source: PLoS Genet. 2013 Jun 6;9(6):e1003514. doi: 10.1371/journal.pgen.1003514 (PMC3674999; doi:10.1371/journal.pgen.1003514)
Supplement: Table S14 — eQTL - target genes associated to the QTL of progenitor cell proliferation in young mice-effect of TGF-beta2 (0.1 ng/ml) on the proliferation of lin-Sca1++kit+ cells . (PDF) [file pgen.1003514.s017.pdf]

Supplementary Table 14. eQTL - target genes associated to the QTL of progenitor cell proliferation in young mice-effect of TGF-beta2 (0.1 ng/ml) on the proliferation of lin-Sca1++kit+ cells [% of ctrl without TGF-beta2].

Target gene	simultaneous FDR	ANOVA FDR	# sign. cond. eQTL	HSC p-value	progenitor cell p-value	erythroid cell p-value	myeloid cell p-value	P-M dynamic eQTL FDR	cis
Jmjd6	0.09035	0.82560	0						no
