# Supplementary material for: Impact of Natural Genetic Variation on Gene Expression Dynamics
Source: PLoS Genet. 2013 Jun 6;9(6):e1003514. doi: 10.1371/journal.pgen.1003514 (PMC3674999; doi:10.1371/journal.pgen.1003514)
Supplement: Table S15 — eQTL - target genes associated to the QTL of proliferative capacity in vitro of bone marrow stem and progenitor cells (lin-Sca1++ c-kit+ cells) in response to KL, flt3L and TPO [number of cells]. (PDF) [file pgen.1003514.s018.pdf]

Supplementary Table 15. eQTL - target genes associated to the QTL of proliferative capacity in vitro of bone marrow stem and progenitor cells (lin-Sca1++ c-kit+ cells) in response to KL, flt3L and TPO [number of cells].

Target gene	simultaneous FDR	ANOVA FDR	# sign. cond. eQTL	HSC p-value	progenitor cell p-value	erythroid cell p-value	myeloid cell p-value	P-M dynamic eQTL FDR	cis
Trappc6a	0.00297	0.45575	0						yes
Napsa	0.00043	0.03846	2	0.95968	< 0.00001	0.07979	< 0.00001		no
Nkg7	0.01528	0.01324	2	< 0.00001	0.00011	0.04501	0.40312		no
Prss16	0.07292	0.19817	0						no
Helb	0.06328	0.22092	0						no
Dmwd	< 0.00001	0.00003	3	0.00018	0.00871	0.00002	< 0.00001		yes
Ctsh	0.05511	0.15702	0						no
Irf8	0.05367	0.82175	0						no
Irf2bp1	< 0.00001	0.66152	0						yes
Opa3	0.00123	0.13122	0						yes
1700008P20Rik	0.06253	0.00006	1	0.32085	1	1	< 0.00001		yes
Vrk2	0.09689	0.00150	1	1	1	< 0.00001	0.03524		no
