# Supplementary material for: Impact of Natural Genetic Variation on Gene Expression Dynamics
Source: PLoS Genet. 2013 Jun 6;9(6):e1003514. doi: 10.1371/journal.pgen.1003514 (PMC3674999; doi:10.1371/journal.pgen.1003514)
Supplement: Table S16 — eQTL - target genes associated to the QTL of transferrin saturation of males and females . (PDF) [file pgen.1003514.s019.pdf]

Supplementary Table 16. eQTL - target genes associated to the QTL of transferrin saturation of males and females [%].

Target gene	simultaneous FDR	ANOVA FDR	# sign. cond. eQTL	HSC p-value	progenitor cell p-value	erythroid cell p-value	myeloid cell p-value	P-M dynamic eQTL FDR	cis
Ndn	0.00010	0.00136	2	< 0.00001	0.00002	0.78380	1		no
Rccd1	0.08524	0.66497	0						no
Olfr998	0.09904	0.76378	0						no
