# Supplementary material for: Impact of Natural Genetic Variation on Gene Expression Dynamics
Source: PLoS Genet. 2013 Jun 6;9(6):e1003514. doi: 10.1371/journal.pgen.1003514 (PMC3674999; doi:10.1371/journal.pgen.1003514)
Supplement: Table S18 — eQTL - target genes associated to the QTL of hemoglobin of 120-day-old females fed 270 ppm iron diet . (PDF) [file pgen.1003514.s021.pdf]

Supplementary Table 18. eQTL - target genes associated to the QTL of hemoglobin of 120-day-old females fed 270 ppm iron diet [μg/dl].

Target gene	simultaneous FDR	ANOVA FDR	# sign. cond. eQTL	HSC p-value	progenitor cell p-value	erythroid cell p-value	myeloid cell p-value	P-M dynamic eQTL FDR	cis
Pdhb	0.05441	0.47086	0						no
Mtmr2	0.04026	0.28556	0						no
Srpr	< 0.00001	0.79846	0						no
Stt3a	0.01654	0.80269	0						no
Dbil5	0.09811	0.91443	0						no
Chmp4b	0.00301	0.82674	0						no
Foxred1	0.00333	0.56419	0						no
Asxl1	0.02960	0.64575	0						no
Ifit2	0.00654	0.54381	0						no
Ddx25	0.79927							0.05918	no
