# Supplementary material for: Impact of Natural Genetic Variation on Gene Expression Dynamics
Source: PLoS Genet. 2013 Jun 6;9(6):e1003514. doi: 10.1371/journal.pgen.1003514 (PMC3674999; doi:10.1371/journal.pgen.1003514)
Supplement: Table S19 — eQTL - target genes associated to the QTL of hemoglobin of 120-day-old females fed 270 ppm iron diet . (PDF) [file pgen.1003514.s022.pdf]

Supplementary Table 19. eQTL - target genes associated to the QTL of hemoglobin of 120-day-old females fed 270 ppm iron diet [μg/dl].

Target gene	simultaneous FDR	ANOVA FDR	# sign. cond. eQTL	HSC p-value	progenitor cell p-value	erythroid cell p-value	myeloid cell p-value	P-M dynamic eQTL FDR	cis
Ccdc14	0.09081	0.28070	0						no
Crip3	0.00670	0.60832	0						no
Kif3b	0.00251	0.48456	0						no
Cdk5rap1	0.03116	0.45068	0						no
Dapk2	0.00066	0.71491	0						no
Cep164	0.01950	0.00024	1	0.74695	1	< 0.00001	1		no
Ly6k	0.08301	0.09780	1	1	1	0.00005	0.33976		no
Sorl1	0.09191	0.07171	2	0.00388	1	0.00005	0.50818		no
Ddx25	0.79927							0.05918	yes
