# Supplementary material for: Impact of Natural Genetic Variation on Gene Expression Dynamics
Source: PLoS Genet. 2013 Jun 6;9(6):e1003514. doi: 10.1371/journal.pgen.1003514 (PMC3674999; doi:10.1371/journal.pgen.1003514)
Supplement: Table S20 — eQTL - target genes associated to the QTL of T cell receptor expression, V-gamma-1 positive, V-gamma-4 positive, of total gamma-delta intestinal intraepithelial lymphocytes . (PDF) [file pgen.1003514.s023.pdf]

Supplementary Table 20. eQTL - target genes associated to the QTL of T cell receptor expression, V-gamma-1 positive, V-gamma-4 positive, % of total gamma-delta intestinal intraepithelial lymphocytes [%].

Target gene	simultaneous FDR	ANOVA FDR	# sign. cond. eQTL	HSC p-value	progenitor cell p-value	erythroid cell p-value	myeloid cell p-value	P-M dynamic eQTL FDR	cis
Epha2	0.01900	0.87082	0						no
2810021B07Rik	0.00043	0.13755	0						yes
Sfrp4	0.03909	0.00410	1	0.01193	< 0.00001	0.48589	1		yes
Trim27	0.06117	0.66459	0						no
Cdk13	< 0.00001	0.00239	3	< 0.00001	< 0.00001	< 0.00001	1		yes
Tnks1bp1	1							0.07424	no
