# Supplementary material for: Impact of Natural Genetic Variation on Gene Expression Dynamics
Source: PLoS Genet. 2013 Jun 6;9(6):e1003514. doi: 10.1371/journal.pgen.1003514 (PMC3674999; doi:10.1371/journal.pgen.1003514)
Supplement: Table S21 — eQTL - target genes associated to the QTL of T cell receptor expression, V-gamma-7 positive and V-gamma-4] positive of total gamma-delta intestinal intraepithelial lymphocytes . (PDF) [file pgen.1003514.s024.pdf]

Supplementary Table 21. eQTL - target genes associated to the QTL of T cell receptor expression, V-gamma-7 positive and V-gamma-4] positive % of total gamma-delta intestinal intraepithelial lymphocytes [%].

Target gene	simultaneous FDR	ANOVA FDR	# sign. cond. eQTL	HSC p-value	progenitor cell p-value	erythroid cell p-value	myeloid cell p-value	P-M dynamic eQTL FDR	cis
Parp2	< 0.00001	0.95191	0						no
6720456H20Rik	< 0.00001	0.00024	3	< 0.00001	0.00001	0.00105	0.02756		yes
Cdkn3	0.00061	0.11999	0						no
1700123O20Rik	< 0.00001	0.39974	0						no
