# Supplementary material for: Impact of Natural Genetic Variation on Gene Expression Dynamics
Source: PLoS Genet. 2013 Jun 6;9(6):e1003514. doi: 10.1371/journal.pgen.1003514 (PMC3674999; doi:10.1371/journal.pgen.1003514)
Supplement: Table S22 — eQTL - target genes associated to the QTL of T cell receptor expression, V-gamma-7 positive and V-gamma-4] positive of total gamma-delta intestinal intraepithelial lymphocytes . (PDF) [file pgen.1003514.s025.pdf]

Supplementary Table 22. eQTL - target genes associated to the QTL of T cell receptor expression, V-gamma-7 positive and V-gamma-4] positive % of total gamma-delta intestinal intraepithelial lymphocytes [%].

Target gene	simultaneous FDR	ANOVA FDR	# sign. cond. eQTL	HSC p-value	progenitor cell p-value	erythroid cell p-value	myeloid cell p-value	P-M dynamic eQTL FDR	cis
Ndrg2	< 0.00001	< 0.00001	3	0.00004	< 0.00001	0	0.89261		yes
Pnp	0.00200	< 0.00001	1	0.70490	0.17439	0	0.47086		yes
Rnase4	0.05418	0.01125	1	0.03600	1	0.10436	< 0.00001		yes
Mcpt8	0.00050	0.50373	0						yes
Psmb5	< 0.00001	0.33292	0						yes
Cebpa	0.08087	0.02803	1	1	0.04757	1	0.00104		no
Apex1	< 0.00001	0.68705	0						yes
Ear11	< 0.00001	< 0.00001	2	0.00973	0.01420	< 0.00001	< 0.00001		yes
Ccnb1ip1	0.00055	< 0.00001	1	0.16137	< 0.00001	0.04095	1		yes
Ang	< 0.00001	0.00105	2	< 0.00001	0.01605	0.02724	< 0.00001		yes
