# Supplementary material for: Impact of Natural Genetic Variation on Gene Expression Dynamics
Source: PLoS Genet. 2013 Jun 6;9(6):e1003514. doi: 10.1371/journal.pgen.1003514 (PMC3674999; doi:10.1371/journal.pgen.1003514)
Supplement: Table S23 — eQTL - target genes associated to the QTL of T cell receptor expression, V-gamma-7 positive, Vgamma-4 negative, of total gamma-delta intestinal intraepithelial lymphocytes . (PDF) [file pgen.1003514.s026.pdf]

Supplementary Table 23. eQTL - target genes associated to the QTL of T cell receptor expression, V-gamma-7 positive, Vgamma-4 negative, % of total gamma-delta intestinal intraepithelial lymphocytes [%].

Target gene	simultaneous FDR	ANOVA FDR	# sign. cond. eQTL	HSC p-value	progenitor cell p-value	erythroid cell p-value	myeloid cell p-value	P-M dynamic eQTL FDR	cis
Dnahc8	0.01568	< 0.00001	1	0.60434	0.73919	1	< 0.00001		no
Lemd2	0.02613	0.02358	1	0.00948	0.65567	< 0.00001	0.21500		no
