# Supplementary material for: Impact of Natural Genetic Variation on Gene Expression Dynamics
Source: PLoS Genet. 2013 Jun 6;9(6):e1003514. doi: 10.1371/journal.pgen.1003514 (PMC3674999; doi:10.1371/journal.pgen.1003514)
Supplement: Table S24 — eQTL - target genes associated to the QTL of T cell receptor expression, V-gamma-7 positive, Vgamma-4 negative, of total gamma-delta intestinal intraepithelial lymphocytes . (PDF) [file pgen.1003514.s027.pdf]

**Supplementary Table 24. eQTL - target genes associated to the QTL of T cell receptor expression, V-gamma-7 positive, Vgamma-4 negative, % of total gamma-delta intestinal intraepithelial lymphocytes [%].**

| Target gene | simultaneous FDR | ANOVA FDR | # sign. cond. eQTL | HSC p-value | progenitor cell p-value | erythroid cell p-value | myeloid cell p-value | P-M dynamic eQTL FDR | cis |
|---|---|---|---|---|---|---|---|---|---|
| *Msh5* | 0.00138 | < 0.00001 | 3 | < 0.00001 | < 0.00001 | 0.47663 | < 0.00001 | | yes |
| *Abhd16a* | < 0.00001 | 0.01441 | 4 | < 0.00001 | < 0.00001 | < 0.00001 | < 0.00001 | | yes |
| *Ddah2* | 0.00048 | 0.11799 | 0 | | | | | | yes |
| *Clic1* | 0.04060 | 0.00383 | 2 | < 0.00001 | 0.00082 | 0.03529 | 1 | | yes |
| *Atf6b* | 0.03295 | 0.10058 | 0 | | | | | | yes |
| *Ppt2* | 0.07559 | 0.00002 | 1 | 1 | 0.86939 | 1 | < 0.00001 | | yes |
| *Wdr46* | < 0.00001 | < 0.00001 | 4 | < 0.00001 | < 0.00001 | < 0.00001 | < 0.00001 | | yes |
| *Vps52* | < 0.00001 | 0.10798 | 0 | | | | | | yes |
| *Ring1* | < 0.00001 | 0.00023 | 4 | < 0.00001 | < 0.00001 | < 0.0000 1 | < 0.00001 | | yes |
| *Psmb9* | < 0.00001 | 0.67772 | 0 | | | | | | yes |
| *Ltb* | 0.00050 | 0.22991 | 0 | | | | | | yes |
| *Ng23* | 0.07523 | < 0.00001 | 2 | < 0.0000 1 | 1 | 1 | < 0.00001 | | yes |
| *H2-Aa* | 0.00031 | < 0.00001 | 3 | < 0.00001 | 0.00677 | < 0.00001 | < 0.00001 | 0.094444 | yes |
| *Vars2* | 0.00050 | < 0.00001 | 2 | < 0.00001 | < 0.00001 | 0.04473 | 0.09614 | | yes |
| *Skiv2l* | 0.07903 | 0.56419 | 0 | | | | | | yes |
| *H2-Ob* | 0.00050 | 0.08208 | 4 | < 0.00001 | < 0.00001 | < 0.00001 | < 0.00001 | | yes |
| *Nfkbil1* | 0.02807 | 0.21138 | 0 | | | | | | yes |
| *Zfp101* | 0.02306 | 0.38374 | 0 | | | | | | yes |
| *H2-K1* | < 0.00001 | 0.00802 | 4 | < 0.00001 | < 0.00001 | < 0.00001 | < 0.00001 | | yes |
| *H2-D1* | < 0.00001 | 0.17514 | 0 | | | | | | yes |
| *Lst1* | 0.06125 | 0.01488 | 2 | 1 | 0.24144 | 0.00050 | < 0.00001 | | yes |
| *H2-Ke6* | 0.09253 | < 0.00001 | 1 | 1 | 1 | 1 | < 0.00001 | 0.08143 | yes |
| *H2-T10* | < 0.00001 | 0.02239 | 4 | < 0.00001 | < 0.00001 | < 0.00001 | < 0.00001 | | yes |
