# Supplementary material for: Impact of Natural Genetic Variation on Gene Expression Dynamics
Source: PLoS Genet. 2013 Jun 6;9(6):e1003514. doi: 10.1371/journal.pgen.1003514 (PMC3674999; doi:10.1371/journal.pgen.1003514)
Supplement: Table S25 — eQTL - target genes associated to the QTL of hematocrit of 120-day-old males and females fed 3 ppm iron diet . (PDF) [file pgen.1003514.s028.pdf]

Supplementary Table 25. eQTL - target genes associated to the QTL of hematocrit of 120-day-old males and females fed 3 ppm iron diet [% packed red blood cells].

Target gene	simultaneous FDR	ANOVA FDR	# sign. cond. eQTL	HSC p-value	progenitor cell p-value	erythroid cell p-value	myeloid cell p-value	P-M dynamic eQTL FDR	cis
Cmpk2	0.01468	0.42402	0						no
Sp100	0.06309	0.16495	0						no
Usp18	0.02601	0.24989	0						no
Oas3	0.06445	0.01479	2	1	1	0.00001	0.00137		no
Oas2	0.07182	0.39974	0						no
Isg15	0.04899	0.62448	0						no
Oasl1	0.03434	0.31480	0						no
4930503L19Rik	0.00031	0.00042	2	0.15435	< 0.00001	< 0.00001	0.51852		yes
Stard6	0.00174	0.10966	0						yes
