# Supplementary material for: Impact of Natural Genetic Variation on Gene Expression Dynamics
Source: PLoS Genet. 2013 Jun 6;9(6):e1003514. doi: 10.1371/journal.pgen.1003514 (PMC3674999; doi:10.1371/journal.pgen.1003514)
Supplement: Table S26 — eQTL - target genes associated to the QTL of thymic T-cell response to anti-CD3-induced proliferation. (PDF) [file pgen.1003514.s029.pdf]

Supplementary Table 26. eQTL - target genes associated to the QTL of thymic T-cell response to anti-CD3-induced proliferation.

Target gene	simultaneous FDR	ANOVA FDR	# sign. cond. eQTL	HSC p-value	progenitor cell p-value	erythroid cell p-value	myeloid cell p-value	P-M dynamic eQTL FDR	cis
Osbp	< 0.00001	0.34326	0						yes
