# Supplementary material for: Impact of Natural Genetic Variation on Gene Expression Dynamics
Source: PLoS Genet. 2013 Jun 6;9(6):e1003514. doi: 10.1371/journal.pgen.1003514 (PMC3674999; doi:10.1371/journal.pgen.1003514)
Supplement: Table S27 — eQTL - target genes associated to the QTL of iron level of plasma of 120-day male and female mice fed 3 ppm iron diet . (PDF) [file pgen.1003514.s030.pdf]

Supplementary Table 27. eQTL - target genes associated to the QTL of iron level of plasma of 120-day male and female mice fed 3 ppm iron diet [μg/dl].

Target gene	simultaneous FDR	ANOVA FDR	# sign. cond. eQTL	HSC p-value	progenitor cell p-value	erythroid cell p-value	myeloid cell p-value	P-M dynamic eQTL FDR	cis
Usp50	0.04343	0.03866	2	1	1	0.00029	0.00239		no
Kcnj12	0.03662	0.44192	0						no
Jrk	0.08009	0.23536	0						no
